# Rational Design of Packaging: Toward Safer and Ecodesigned Food Packaging Systems

**DOI:** 10.3389/fchem.2019.00349

**Published:** 2019-05-15

**Authors:** Yan Zhu, Bruno Guillemat, Olivier Vitrac

**Affiliations:** ^1^UMR GENIAL, INRA, AgroParisTech, Université Paris-Saclay, Massy, France; ^2^Pernod Ricard—Breakthrough Innovation Group, Paris, France

**Keywords:** ecodesign, packaging, mass transfer, alcoholic beverages, shelf-life, polyethylene terephthalate, thermodynamics

## Abstract

It is necessary and urgent to reduce the impact of single-use plastics and in particular those used for beverages and similar products. Fewer plastics or reduced shelf-life need to be explored simultaneously while keeping open the possibilities of bringing on the innovative market designs and consumer experiences. This work details a general computational framework bridging simulation of coupled mass transfer and non-linear thermodynamics with 3D geometry optimization, ecodesign and other mass transfer-related risks (collapse, migration of chemicals). The three-dimensions (impacts, design, shelf-life) of the storage of alcoholic beverages in plastic containers are presented as a representative case-study of the multi-objective ecodesign problem and as an illustration of new trends to accelerate prototyping and innovation. The capacity to evaluate, maximize product shelf-life and minimize packaging waste was tested on 450 miniatures served usually in plane and train. Four alcoholic strengths (including pure water) and four storage conditions were considered. Several alternatives (900 different 3D designs) were explored by simulation and constrained optimization to reach the targeted shelf-life. The large set was used to establish the Pareto optimal design enabling to minimize weights while maximizing shelf-life. The approach is sufficiently versatile and flexible to adapt arbitrary designs and additional phenomena controlling shelf-life (aroma scalping, oxidation, migration of non-intentionally added substances).

## Introduction

During the last decade, the perception of plastics by the public and authorities has fundamentally evolved from beneficial to significantly negative (Hamaide et al., 2014). They have been successfully associated with potential sources of harmful substances, wastes, greenhouse gases, etc. The negative perception has been amplified by the absence of harmonized regulations (see an overview of regulations in the world in Rijk and Veraart, 2010) and the lack of good manufacturing and design practices for materialsused in association in packaging components (laminates, printed and coated surfaces, closure with gaskets or varnishes, plastics associated with paper and board…). Among developed countries, the EU is equipped with the strictest arsenal of rules. EU framework regulation 1935/2004/EC (EC, 2004b) makes a risk assessment and risk management compulsory for the introduction of any new substance, material or industrial practice (active/intelligent packaging system, recycling) regardless the material is in plastics or not. While not mentioning design, EU regulation 2023/2006/EC (EC, 2006) encourages the developments of good manufacturing practices and quality assurance systems at all stages of the production and handling for each of the 17 groups materials and their combinations accepted for food contact. The reduction of the environmental impact of packaging and its wastes has been enforced since 25 years ago in EU with the Directive 94/62/EC (EEC, 1994) and its successive amendments (EC, 2004a, 2005, 2015). Its annex II imposes that any “packaging shall be so manufactured that the packaging volume and weight be limited to the minimum adequate amount to maintain the necessary level of safety, hygiene, and acceptance for the packed product and for the consumer.” Since foods are not specifically mentioned, no association has been made between barrier properties, shelf-life, and amount of plastic materials and finally wastes. Food packaging represents in western countries most of the plastic wastes (Plastics Europe, 2018) with a growing contribution of ready-to-eat foods (Silberbauer and Schmid, 2017). A recent study (Wilson et al., 2017) showed that the willingness of consumers to waste food was affected not only by product size and expiration dates but also by date labels themselves. In simple words, reducing the shelf-life of food products would enable the use of less barrier materials, but will also increase food waste and will reduce access to global markets. FAO (Gustavsson et al., 2011) concluded that well-designed packaging was strongly effective to reduce food losses and waste for all food categories and all along the food supply chain until the final consumer. As a result, the design of a new packaging for a new product or market should be envisioned as a trade-off between several issues: safety, shelf-life and environmental impact.

This study addresses food packaging design as a multicriteria optimization problem, which can be tackled with the tools and concepts of Chemical Engineering used to reduce material and energy requirements (Rangaiah and Bonilla-Petriciolet, 2013). For food packaging, the chief question is how to maximize shelf-life while minimizing packaging weight in worst-case storage and transport conditions (min-max problem). Additional problems of minimization, such as mitigating migration issues of intentionally-added substances, can also be considered during packaging design as shown by Nguyen et al. (2013). The considered degrees of freedom are various, they include the choice of the materials, their formulation and industrial practices along the supply chain; but only the packaging shape and wall thicknesses are common to all mass transfer problems: mass loss, migration issues, aroma scalping, oxidation, etc. The proposed approach is complementary to life cycle assessment (LCA) codified in standard ISO 14040-2006 (ISO, 2006), which can be used to recommend materials, but which lacks enough granularity to orient packaging design. Bottles in polyethylene terephthalate (PET) offer the best illustrative example of environmental challenges, as they represent 7.4 % of all packaging (Plastics Europe, 2018) and have been associated with the growing marine litter in oceans and seas (Jambeck et al., 2015). For comparison, one kilogram of glass is approximately required to produce two bottles of one-liter capacity when 28 bottles can be processed with one kilogram of PET. When PET bottles are produced from recycled material, as authorized in EU regulation 282/2008/EC (EC, 2008a), they offer low environmental footprints (Toniolo et al., 2013; Accorsi et al., 2015; Manzardo et al., 2016). Since the shelf-life of pasteurized, sterilized, and liquid food products tend to be shortened in plastic bottles, the food product and its convenience should be introduced in the holistic approach. Similar conclusions were drawn by Wikström et al. (2014) by noticing that that food production has a higher impact on the environment than the packaging. For single-use beverage bottles, the draft of EU directive 2018/0172/EC (EC, 2018) on the reduction of the impact of certain plastic products on the environment favors product design requirements too restrictive and awareness measures. Original LCA methodology is mainly forensic and retrospective. It has therefore little applicability at early stages of product development when the concepts do not enable a clear determination of material use, energy consumption, and impacts (Holdway et al., 2002). Despite 29 methods available for sustainable product development (Buchert et al., 2017), current global indices remain too rough and too uncertain to finalize design details (Yu et al., 2017). Rezaei et al. (2019) proposed a pairwise preference comparison methodology recently to select the best tradeoff between the consumer acceptability and environment impacts. The approach is qualitative and does not integrate the food explicitly. During the last decades, life cycle design and ecodesign have been promoted by authorities (Keoleian and Menerey, 1993; EC, 2018) and international organizations (ISO, 2002; UNEP, 2009). They are considered as pioneering concepts for food packaging design (Karlsson and Luttropp, 2006; González-García et al., 2016). The methods and tools of ecodesign are, however, still at a primitive stage and can be considered as “vectors of learning, i.e., allowing designers to improve their individual and collective expertise in eco-design” (Vallet et al., 2013). In details, they exhibit some similarities with the TRIZ-problem solving method (Ilevbare et al., 2013) by reusing a knowledge base to self-similar problems: renewable or recycled materials, low material weight, efficient transport, reusability, recyclability or compostability, low waste emissions, etc. Innovative solutions integrating original design, consumer convenience, and new functionalities cannot be explored, because mainly engineering aspects are not explicitly considered such as packaging weight, shape, barrier properties, food shelf-life, cost, safety, etc. By taking the example of alcoholic beverages stored in PET bottles as a representative case study, a tailored and more systematic methodology is proposed to solve, holistically, several mass transfer problems impacting the amount of wastes, the shelf-life of the product and food safety issues. The developments must be seen as an extension of safe-by-design concepts already applied to food packaging (Nguyen et al., 2013). The proposed multiscale modeling, simulation, and optimization framework reuses indeed several mass transfer simulation components of the open-source project FMECAengine (Vitrac, 2018). The chained simulation engine and the procedural solver enable was initially developed to offer concurrent engineering on contamination issues along the supply chain and between packaging components for one to several hundreds of substances. The new extensions focus on packaging geometry optimization in the presence of coupled mass transfer (the diffusion of one substance is affecting the others). Additionally, the new capability to integrate mass transfer equations over three dimensional geometry can be seen complementary to 3D simulation-optimization strategies aiming at maximizing mechanical resistance of bottles with less plastic material (Masood and Keshavamurthy, 2005; Demirel and Daver, 2009; Daver et al., 2012; Hu et al., 2012; Huang et al., 2018). Mass transfer are, indeed, rarely considered in such approaches (Abbès et al., 2010) and never while taking into account the impact on the shelf-life of the food product inside.

The paper is organized as follows. The principles of the description of coupled mass transfer of water and ethanol across polymer walls are described in section theory. Special attention is devoted to the thermodynamical coupling between the chemical potential of water and ethanol in the case of alcoholic beverages. The consequences of mass transfer on the total pressure inside the packaging, the primary cause of leaks and packaging collapse, are also discussed according to packaging filling conditions. Section materials and methods details studied experimental conditions and our strategy of 3D simulation under geometry and shelf-life constraints. The thermodynamical diagrams and the global validation of our predictive approaches are presented in section results and discussion for PET miniatures, which are small plastic bottles containing fruit juices, liquors, or other alcoholic beverages, usually served in trains and planes, and also available in hotel minibars. Comparatively to full-size bottles, their design, and their conditions of storage are very challenging both (i) for the shelf-life of the beverage, as they have the highest surface-to-volume ratios packaging in particularly, and (ii) for the constraints of weight at the board of an aircraft. This example is used to demonstrate that conflicting constraints packaging weight and shelf-life can be managed in a consensual manner if the first is correctly adjusted to the second and if new shapes are introduced. Though only coupled water and ethanol mass transfer from the inside to the outside of the packaging are considered in this study, the approach remains transposable to any permeation, and migration problem met in beverages, pharmaceutical, cosmetic, and biotechnological products. The main findings are summarized in the last section.

## Theory

### Shelf-Life of Alcoholic Beverages

Packaging for long shelf-life products is designed to offer significant barrier properties to gases from the ambiance (water vapor, oxygen) and to food constituents. Aside from oxidation issues in oxygen-sensitive products, the reduction of shelf-life of beverages is primarily caused by mass loss (usually water content) and by the loss of both aroma and sapid compounds. Weight loss occurs as the balance of all mass transfer across container walls and the closure system. As an example, water bottles stored in a dry place are losing weight due to a net permeation rate of water from the inside to the outside. The loss of aroma is usually slower and associated to reversible sorption in the walls, coined “scalping” process (Ducruet et al., 2007; Dombre et al., 2015); and which dominates over mass loss by permeation. Predicting the rates of each mass transfer requires a proper characterization of diffusion and sorption properties in the considered polymer, usually a polyester material such as polyethylene terephthalate (PET), at the different stages of transportation, retailing, storage, and final consumption. In this study, the non-linear behavior of water-ethanol mixtures stored in PET bottles is thought to represent the properties of real alcoholic beverages such as ciders, beer, wines, and spirits realistically. Since only water and ethanol are considered, the type of beverage is defined by its alcoholic strength by volume, denoted abv. It is legally defined in EU as “the ratio of the volume of pure alcohol present in the product in question at 20°C to the total volume of that product at the same temperature” (see Annex I of EU Regulation 110/2008; EC, 2008b). The case of bottled water can be seen as a special case corresponding to abv = 0. Tolerances setting the shelf-life of alcoholic beverages are also relative to the variation of abv during storage and transportation, denoted |Δ*abv*|. EU regulation 1169/2011/EC (EC, 2011) sets out a stringent tolerance of ± 0.3% for non-beer related beverages with abv values larger than 1.2%. Finally, the EU applies a tolerable negative error of 1.5% for the weight of prepackaged liquids, Δ*w*, with volumes equal or larger than 1 L (see Annex I of Directive 76/211/EEC; EEC, 1976). In the context of plastic containers, it should be interpreted as the maximum allowable variation from the weight labeled on the package according to unavoidable variations in weighing, measuring, and mass transfer across packaging walls.

### Common Assumptions

Weight and abv tolerances are governed by the mass transfer of both water and ethanol, with possible antagonist effects. For instance, losing water in a dry storage place will concentrate ethanol and increase consequently abv. Losing simultaneously water and ethanol will, by contrast, keep constant abv or will make it decrease. The effects are highly non-linear and require (i) a proper description of the thermodynamics of water-ethanol mixtures during the shelf-life of the beverage and (ii) a predictive description of mutual sorption and diffusion phenomena across the walls of the container. In a real container, the liquid mixture is in equilibrium with a headspace containing air or an inert gas. Its volume and its pressure are complex functions of temperature (thermal expansion of the liquid and gas in fact), of mechanical constraints applied on the semi-flexible packaging, but also of the resulting mass transfer between the liquid and its headspace. This section introduces a comprehensive and consistent description for arbitrary water-ethanol mixtures below the boiling point of ethanol, enabling to predict overpressure and collapse issues. Mass transfer across the walls of the container are described in simplified but not oversimplified calculations, which do not require any steady state for the fluxes for water and ethanol, but which neglect the modifications of the polymer induced by the sorption of water and ethanol. In the case of PET, polymer relaxation effects are triggered by critical temperatures and relative humidities (see the pseudo state diagram 5 in Dubelley et al., 2017), but they do not modify the diffusion coefficients of water in PET significantly (see Figure 14 in Burgess et al., 2014a). Although such non-linear behaviors could have been integrated, it has been preferred to use robust simulations based on worst-case assumptions, which enable the full exploration of the geometry space for containers intended to preserve the properties of alcoholic beverages under strict tolerance constraints (|Δ*abv*| ≤ 0.3% and Δ *w* ≥ −1.5%), while minimizing the amount of plastic material to produce the container itself.

### Thermodynamics of Alcoholic Beverages

Water and ethanol mass transfer, denoted with subscripts *w* and *e*, are controlled by two driving forces: partial pressure gradients across walls and the difference of total pressure on both sides of the closure system. By neglecting internal mass transfer in the beverage and the heat transfer required for the vaporization or the condensation of water and ethanol at the liquid-gas interface, the driving potentials on the beverage side are set by the total pressure and the partial pressures in the headspace.

#### Practical Approximations of Partial Pressures: ${\left\{p_{i}^{\left(T\right)}\right\}}_{i=e,w}$

In this study, the state of pure-components is chosen as reference state of water and ethanol. As a result, the partial pressures, ${\left\{p_{i}^{\left(T,abv\right)}\right\}}_{i=e,w}$in the headspace, can be inferred from the binary activity coefficients, ${\left\{\gamma_{i}^{\left(T,abv\right)}\right\}}_{i=e,w}$, relative to the molar fraction of water or of ethanol in the beverage, {_*x*_*i*_}*i* = *e, w*_ and from their pure vapor saturation pressures ${\left\{p_{i,sat}^{\left(T\right)}\right\}}_{i=e,w}$:

(1) $$\begin{array}{l} p_{i}^{\left(T,abv\right)}=x_{i}\gamma_{i}^{\left(T,abv\right)}p_{i,sat}^{\left(T\right)}\text{for }i=e,w \end{array}$$

with *T* the absolute temperature in Kelvin, but expressed in °C in the description of storage conditions.

Equation (1) is exact when the interactions between water (and also ethanol in a less extent) and the other beverage constituents (pectins, proteins, aroma…) can be neglected. When the real alcoholic beverage is replaced by an equivalent hydroalcoholic solution, one gets:

(2) $$\begin{array}{l} x_{e}=1-x_{w}\;for\;i=e,w \end{array}$$

By noticing that *abv* is defined at 20°C, molar fractions are related to the density of the mixture with the same composition at the same temperature, denoted $\rho_{w+e}^{\left(20{}^{\circ}C,abv\right)}$:

(3) $$\begin{array}{l} x_{e}=\frac{w_{e}/M_{e}}{w_{e}/M_{e}+\left(1-w_{e}\right)/M_{w}}\\ \;=\frac{\frac{1}{M_{e}}\left(\frac{\rho_{e}^{20{}^{\circ}C}}{\rho_{w+e}^{\left(20{}^{\circ}C,abv\right)}}abv\right)}{\frac{1}{M_{e}}\left(\frac{\rho_{e}^{20{}^{\circ}C}}{\rho_{w+e}^{\left(20{}^{\circ}C,abv\right)}}abv\right)+\frac{1}{M_{w}}\left(1-\frac{\rho_{e}^{20{}^{\circ}C}}{\rho_{w+e}^{\left(20{}^{\circ}C,abv\right)}}abv\right)} \end{array}$$

with $w_{e}=\frac{\rho_{e}^{20{}^{\circ}C}}{\rho_{w+e}^{\left(20{}^{\circ}C,abv\right)}}abv$ being the weight fraction in ethanol, {*M*_*i*_}_*i* = *e, w*_ the molecular weights and ${\left\{\rho_{i}^{20{}^{\circ}C}\right\}}_{i=e,w}$ the densities of pure components at 20°C.

#### Practical Approximation of the Total Pressure in the Headspace in Equilibrium With the Beverage.

The total pressure in the headspace *P*_*head*_ results from several equilibria: (i) the chemical equilibrium between the liquid and the headspace, (ii) the thermal equilibrium with the gas and liquid phase and (iii) the balance of forces on the sides of the wall. All equilibria are connected, changing temperature or changing the volume of the headspace affect both the total pressure and the composition of the headspace (and corollary the composition of the liquid). The presence of air or an inert gas affect these equilibria. An efficient procedure to calculate the final total pressure and composition of the headspace has been devised. It takes into account the internal volume of the bottle *V*_*bottle*_, the initial temperature *T*_0_, the initial total pressure *P*_0_, the initial composition of the beverage *abv*_0_ and the initial headspace volume $V_{head}^{t=0}$. Without a loss of generality, some simplifications are introduced. The deformations of the wall are not considered (the total volume of the gas and liquid is constant), the leaks and mass transfer across the walls are discarded (the equilibration is faster than permeation), the headspace behaves as an ideal gas, air or inert gas cannot dissolve in the liquid and is incondensable.

At any time *t*, the number of solutes *i* = *w, e* in the gas phase, ${\left\{n_{i}^{g}\right\}}_{i=w,e}$, is derived by combining Equation and the ideal gas law as:

(4) $$\begin{array}{l} n_{i}^{g}=p_{i}\frac{V_{head}}{RT}=\gamma_{i}^{\left(T,abv\right)}x_{i}\frac{P_{i,sat}^{\left(T\right)}V_{head}}{RT}. \end{array}$$

where *V*_*head*_ is the volume of headspace and *R* is the ideal gas constant.

Since air (or the inert gas) is assumed to be incondensable, insoluble and initially dry (no water inside), the number of air molecules, *n*_*a*_, is given by the initial amount of air in the headspace:

(5) $$\begin{array}{l} n_{a}=\frac{P_{0}V_{head}^{t=0}}{RT_{0}}. \end{array}$$

Equation (5) and subsequent mass balance on air assumes that nitrogen and oxygen are not exchanged between the beverage and the headspace. The assumption is reasonable with nitrogen (low solubility), but questionable for oxygen. At atmospheric conditions, the solubility of oxygen in water is very low, about eight parts of oxygen per million at 25°C (Truesdale and Downing, 1954). Its solubility increases slightly with ethanol content to reach a value of 3.5% higher than in pure water for *abv* = 0.15(see Table 1 and Figure 1 of Kutsche et al., 1984). The total desorption or sorption of oxygen in a headspace representing 1:15 of the volume beverage will cause a variation of the total pressure lower than ±0.02 %. Comparatively to the risk associated with ethanol and water, the contribution of oxygen on the risk of collapse was therefore neglected. In the absence of an inert gas, the beverage is additionally assumed to be already at or close to the equilibrium with the earth atmosphere, so that significant desorption, or sorption of oxygen is unlikely.

By assuming that the headspace does not contain ethanol initially before the bottle is filled, the total number of ethanol molecules in the bottle is hence given by:

(6) $$\begin{array}{l} n_{e}^{t=0}=\frac{\rho_{e}^{20{}^{\circ}C}}{M_{e}}\left(V_{bottle}-V_{head}^{t=0}\right)abv_{0}. \end{array}$$

For water, the number of water molecules in the bottle is derived from the density of the initial water-ethanol mixture, $\rho_{w+e}^{\left(T_{0},abv_{0}\right)}$:

(7) $$\begin{array}{l} n_{w}^{t=0}=\frac{\rho_{w+e}^{\left(T_{0},abv_{0}\right)}\left(V_{bottle}-V_{head}^{t=0}\right)-n_{e}^{t=0}M_{e}}{M_{w}}. \end{array}$$

For a given volume of the headspace *V*_*head*_, the theoretical density of the liquid mixture, $\rho_{w+e}^{theoretical}\left(V_{head}\right)$, is given:

(8) $$\begin{array}{l} \rho_{w+e}^{theoretical}\left(V_{head}\right)=\frac{\left(n_{e}^{t=0}-n_{e}^{g}\right)M_{e}+\left(n_{w}^{t=0}-n_{w}^{g}\right)M_{w}}{V_{bottle}-V_{head}}. \end{array}$$

Equilibrium is reached when the density of the mixture matches the equilibrium density of hydroalcoholic mixture as reported in handbooks and denoted $\rho_{w+e}^{\left(T,abv\right)}$. Finding *V*_*head*_ is equivalent to solve the equality:

(9) $$\begin{array}{l} \rho_{w+e}^{theoretical}\left(V_{head}\right)=\rho_{w+e}^{T}\left(x_{e}\left(V_{head}\right)\right). \end{array}$$

with:

(10) $$\begin{array}{l} x_{e}\left(V_{head}\right)=\frac{n_{e}^{t=0}-n_{e}^{g}\left(V_{head}\right)}{n_{e}^{t=0}+n_{w}^{t=0}-n_{e}^{g}\left(V_{head}\right)-n_{w}^{g}\left(V_{head}\right)}. \end{array}$$

Finally, the total pressure in the headspace at equilibrium is given by:

(11) $$\begin{array}{l} P_{head}=\frac{n_{a}+n_{w}^{g}+n_{e}^{g}}{V_{head}}RT \end{array}$$

The full procedure reads:

(12) $$\begin{array}{l} \begin{array}{c} 1:\text{ calculate }n_{a}\left(Eq.7\right),n_{e}^{t=0}\left(Eq.8\right),n_{w}^{t=0}\left(Eq.9\right)\\ 2:\text{ initialization }V_{head}\leftarrow V_{head}^{t=0}\text{ at }T_{0}\\ 3:\text{ initialization }x_{e}\leftarrow x_{e}^{t=0}\\ 4:x_{e}^{old}\leftarrow x_{e}^{t=0},\text{ calculate }{\left\{n_{i}^{g}\right\}}_{i=w,e}\left(Eq.6\right)\\ \text{with the previous guess of }x_{e},1-x_{e}\\ 5:\left(\text{fix point iteration}\right):\text{ update }x_{e}\left(Eq.12\right)\\ 6:\text{ go to step 4 until }|x_{e}-x_{e}^{old}|\leq \\ 7:\text{ find }abv\text{ from }x_{e}\left(Eq.3\right)\text{ and read }\rho_{w+e}^{\left(T,abv\right)}\\ 8:\text{ calculate }\rho_{w+e}^{theoretical}\left(V_{head}\right)\left(Eq.10\right)\\ 9:\text{ calculate }\Delta \rho =|\rho_{w+e}^{\left(T,abv\right)}-\rho_{w+e}^{theoretical}\left(V_{head}\right)|\\ 10:\text{ update }V_{head}\text{ according to the Gold section search method}\\ \text{11: go to step 3 until }\Delta \rho \text{ is minimized}\\ \text{12: calculate }P_{head}\left(Eq.13\right) \end{array} \end{array}$$

### Transport Equations at Bottle Walls

The internal side of the container (e.g., bottle) is exposed to the vapors of the water-ethanol mixture whereas the external side is exposed only to the relative humidity of the storage place or room at the same temperature. In this description, the beverage is assumed to be macroscopically at thermal equilibrium with the surroundings and that the storage place does not accumulate ethanol (i.e., good ventilation and extraction).

Without distinguishing whether the mass transfer was initiated in the liquid or in the headspace compartment (they are both at thermodynamical equilibrium), the diffusive fluxes at the interfaces in contact with polymer walls are described satisfactorily within the thin film approximation. On the internal side, denoted ∂Ω_int_ and its normal vector **n**_∂_Ω__int__, an effective mass transfer resistance can be considered to account for the vaporization-condensation process associated with the sorption of water and ethanol. By denoting ${\left\{C_{i}^{x,y,z,t}\right\}}_{i=e,w}$ the solute concentration (SI units in kg·m^−3^) in the walls at a position (*x, y, z*) at the time *t* and ${\left\{f_{i,P}^{T}\right\}}_{i=e,w}$the binary sorption isotherms in the walls, the mass flux densities at the internal boundary condition reads:

(13) $$\begin{array}{l} \left[\begin{array}{l} j_{e}^{\left(T,abv\right)}{|}_{\partial \Omega_{\text{int}}}\\ j_{w}^{\left(T,abv\right)}{|}_{\partial \Omega_{\text{int}}} \end{array}\right]=\left[\begin{array}{l} -D_{e}^{\left(T\right)}\nabla C_{e}^{x,y,z,t}{|}_{\partial \Omega_{\text{int}}}\cdot \;n_{\partial \Omega_{\mathrm{int}}}\\ -D_{w}^{\left(T\right)}\nabla C_{w}^{x,y,z,t}{|}_{\partial \Omega_{\text{int}}}\cdot \;n_{\partial \Omega_{i\text{nt}}} \end{array}\right]\\ \;=\left[\begin{array}{l} h_{e}\frac{p_{e,sat}^{\left(T\right)}}{M_{e}RT}\left(x_{e}\gamma_{e}^{\left(T,abv\right)}-f_{e,P}^{T}-1\left(C_{e}^{x,y,z,t}{|}_{\partial \Omega_{\text{int}}}\right)\right)\\ h_{w}\frac{p_{w,sat}^{\left(T\right)}}{M_{w}RT}\left((1-x_{e})\gamma_{w}^{\left(T,abv\right)}-f_{e,P}^{T}-1\left(C_{w}^{x,y,z,t}{|}_{\partial \Omega_{\text{int}}}\right)\right) \end{array}\right] \end{array}$$

where ${\left\{{f_{i,P}^{T}}^{-1}\right\}}_{i=e,w}$is the inverse function of the function ${\left\{f_{i,P}^{T}\right\}}_{i=e,w}$giving the amount of solute *i* absorbed in the walls according to its activity at temperature *T*; {_*h*_*i*_}*i* = *e, w*_ is the equivalent mass transfer conductance across the boundary layer (SI units in m·s^−1^). The molar fraction of ethanol in the mixture *x*_*e*_ is derived from its weight fraction $\frac{m_{e}^{\left(t\right)}}{m_{e}^{\left(t\right)}+m_{w}^{\left(t\right)}}$ (see Equation 3) and the global mass balance in water and ethanol over all internal interfaces of the packing. For a bottle of height *H* and cylindrical symmetry, the rates of variation of the mass in water and ethanol, denoted $m_{w}^{\left(t\right)}$ and $m_{e}^{\left(t\right)}$, reads:

(14) $$\begin{array}{l} \frac{d}{dt}\left[\begin{array}{l} m_{e}^{\left(t\right)}\\ m_{w}^{\left(t\right)} \end{array}\right]=\underset{\partial \Omega_{{}_{\text{int}}}}{∯}\left[\begin{array}{l} j_{e}^{\left(T,abv,x,y,z\right)}{|}_{\partial \Omega_{\text{int}}}\\ j_{w}^{\left(T,abv,x,y,z\right)}|{|}_{\partial \Omega_{\text{int}}} \end{array}\right]dA\\ \;=\int_{z=0}^{H}\left[\begin{array}{l} j_{e}^{\left(T,abv,z\right)}\\ j_{w}^{\left(T,abv,z\right)} \end{array}\right]2\pi r\left(z\right)dz \end{array}$$

where *r*(*z*) is the radial profile of the bottle along the vertical coordinate *z*.

### Transport Equations Within Bottle Walls and Strategy of Resolution

Since the thickness of bottle walls is several magnitude orders smaller than the height of the bottle, mass transfer through bottle walls can be therefore assumed one-dimensional without a significant loss of accuracy. By neglecting the contribution of the polymer relaxation as driving force, the mutual diffusion of water and ethanol in polymer walls reads:

(15) $$\begin{array}{l} \frac{\partial }{\partial t}\left[\begin{array}{c} C_{e}^{\left(r,z,t\right)}\\ C_{w}^{\left(r,z,t\right)} \end{array}\right]=\frac{1}{r}\frac{\partial }{\partial r}(r\left[\begin{array}{cc} D_{e}^{\left(T,[C_{e},C_{w}{]}^{\prime },z\right)} & 0\\ 0 & D_{w}^{\left(T,[C_{e},C_{w}{]}^{\prime },z\right)} \end{array}\right]\\ \frac{\partial }{\partial r}\left[\begin{array}{c} C_{e}^{\left(r,z,t\right)}\\ C_{w}^{\left(r,z,t\right)} \end{array}\right]) \end{array}$$

where ${\left\{D_{i}^{\left(T,C_{i},z\right)}\right\}}_{i=w,e}$ is the diffusion coefficient in the normal direction of the walls. The dependence with the vertical position *z*and with the local solute concentration accounts for non-uniform drawing and plasticizing effects.

Equation (15) can be solved efficiently for any arbitrary initial solution ${\left[C_{e}^{\left(r,z,t=0\right)},C_{w}^{\left(r,z,t=0\right)}\right]}^{{}^{\prime }}$ along with boundary condition by decomposing the bottle into vertical sections with similar thickness and transport properties. In other words, mass transfer across container walls can be factorized into *n* ≥ 1 boundary conditions between the beverage and *n* equivalent sections coupled via mass balance Equation. By assuming that container walls are at equilibrium with the storage atmosphere at a relative humidity *RH*_0_ and a temperature *T*_0_, and which is not contaminated by ethanol vapors, the corresponding initial condition is:

(16) $$\begin{array}{l} \left[\begin{array}{c} C_{e}^{\left(r,z,t=0\right)}\\ C_{w}^{\left(r,z,t=0\right)} \end{array}\right]=\left[\begin{array}{c} 0\\ f_{w,P}^{T_{0}}\left(RH_{0}\right) \end{array}\right] \end{array}$$

Equations (1–3) (13–6) have been implemented by following the finite-volume formulation of Nguyen et al. (2013) and the opensource project FMECAengine (Vitrac, 2018).

## Materials and Methods

### Mass Transfer Challenge Test on Real Bottles

**Figure 1:**
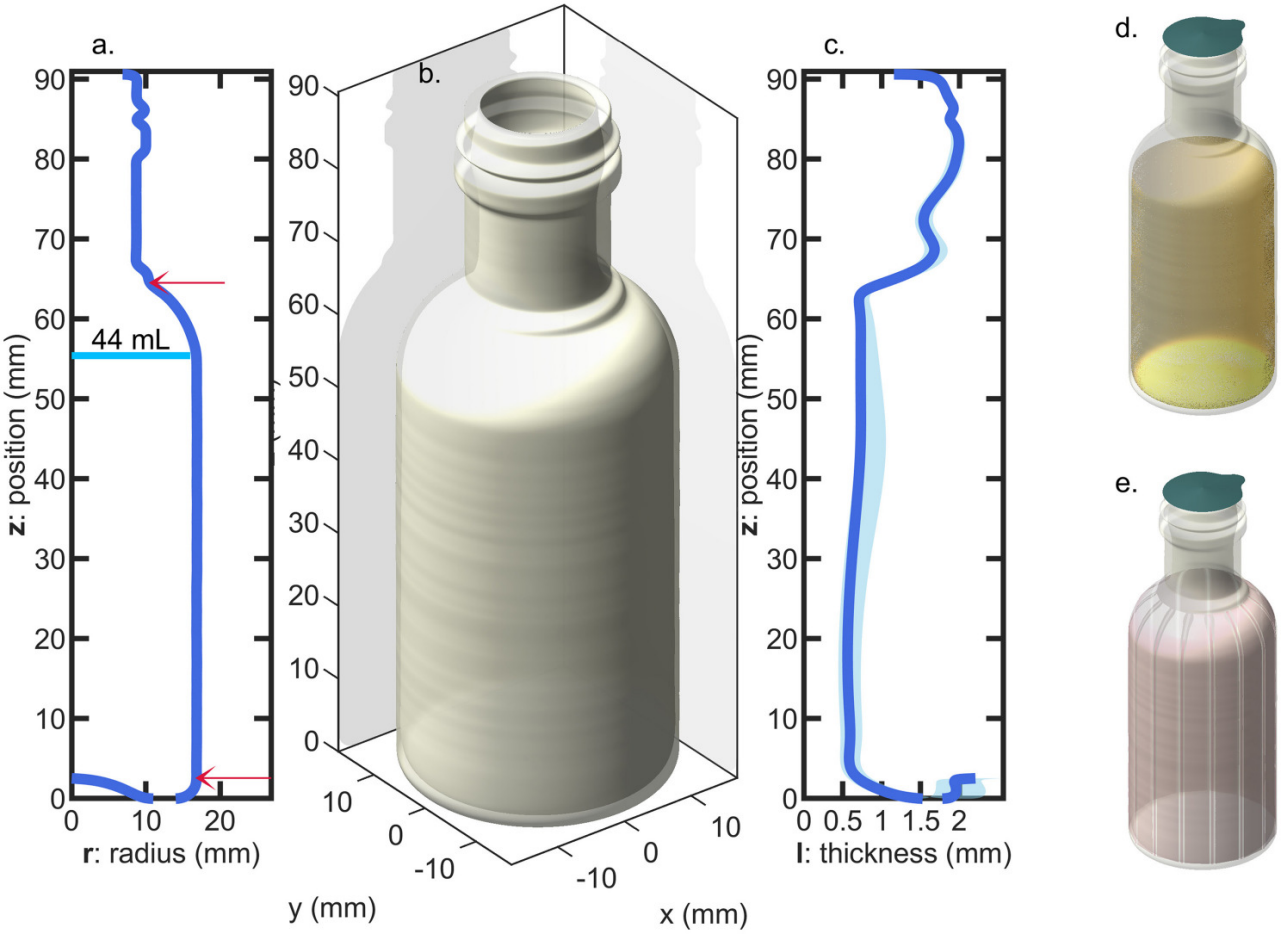
Bottle geometry: **(a)** radius profile (the arrows indicate the top and bottom position of the sleeve and the vertical line indicate the level of liquid for pure water); **(b)** 3D-representation assuming a revolution geometry; **(c)** thickness profile (the shadow indicates the variation range of measurements among 10 bottles); **(d)** filled and sealed bottle; **(e)** bottle equipped with a shrink sleeve label.

Miniature PET bottles with a capacity of 55 mL (injected-mold, supplier Pernod Ricard, France) and a weight of 10.4 ± 0.2 g were used to carry out a long-term challenge test mimicking real storage conditions (up to 7 months) and real alcoholic typical beverages. The bottles were produced by one step injection-blowing. The crystallinity of main bottle walls was determined by the differential scanning calorimetry (model DSC 3, METTLER TOLEDO) and estimated to 32 ± 1.2% (based on three bottles), with an enthalpy of fusion of 140.1 J·g^−1^ as reported by Wunderlich (2005). The geometry of the bottles and the profile of wall thickness are shown in Figure 1. The thickness profile was measured in more than fifty different random locations per bottles on 10 randomly sampled bottles with the help of a magnetic wall thickness gauge (model MiniTest 7,200 FH with probe FH10, ElektroPhysik GmbH, Germany).

Four hundred twenty-five flasks were tested in conditions simulating four typical beverages listed in Table 1 and severe storage conditions reported in Table 2. Two temperature levels (35 and 50°C), three levels of relative humidity, RH, (10, 20, and 75%) and four *abv* levels (0, 0.15, 0.4, 0.7) were covered. Temperatures ± 0.5°C and relative humidities ± 2% were controlled and monitored in three climatic chambers with renewed air. Bottles were filled with 44 ± 0.5 mL at room temperature and atmospheric pressure to keep the headspace volume independent of the hydroalcoholic solution considered. They were subsequently and without delay sealed with 21.5 mm diameter pre-cut foils (aluminum-polyester lid, supplier Embatherm, France) as shown in Figure 1d. A lab-scale pneumatic-controlled heat sealer (model TIME 160, Embatherm, France) was used with a set temperature of 180°C and a set pressure of 200 kPa. Large headspace was preferred to minimize the overpressure and minimize the risk of leaking. Under these specifications, the number of leaking bottles was ranged from 40 (for the most severe condition 1) down to 0 (for pure water stored in all conditions). The initial test was prolonged for beverages B3 and B4 stored in condition S4 by adding a 60 μm thick shrinkable PET sleeve label (supplier Fuji Seal France SAS, France) on remaining bottles as illustrated in Figure 1d. The sleeve did not cover the whole surface area of the bottle (positions are indicated in Figure 1a), but it was sufficient to induce a mass transfer reduction via the thinner surface areas and to confirm the physical description of mass transfer by mutual sorption/permeation across the walls of the bottles.

**Table 1:** Hydroalcoholic solutions simulating real alcoholic beverages.

**Beverage code**	**abv _**target**_ (–)**	**abv _**measured**_ (–)**	**Comment**	**Number of bottles and measurements in each storage condition^**†**^:**			
				**S1**	**S2**	**S3**	**S4**
B1	0.7	0.7004 ± 9 × 10^−4^	simulant of absinthe type	a. 84	a. 82	a. 77	a. 10
				b. 44	b. 57	b. 64	b. 0
				c. 247	c. 265	c. 318	c. 9
				d. 45	d. 44	d. 59	d. 0
B2	0.4	0.4029 ± 6 × 10^−4^	simulant of vodka type	n.c.	n.c.	a. 85	n.c.
						b. 66	
						c. 338	
						d. 66	
B3	0.15	0.1515 ± 5 × 10^−4^	simulant of wine type	n.c.	n.c.	a. 85	n.c.
						b. 77	
						c. 393	
						d. 72	
B4	0	0	deionized water	a. 10	a. 10	a. 30	a. 10
				b. 0	b. 0	b. 0	b. 0
				c. 57	c. 62	c. 209	c. 180
				d. 0	d. 0	d. 0	d. 0

† *see the storage condition code in Table 2*.

*a. total tested bottle; b. non-leaking bottles; c. mass loss measurement; d. abv measurement*.

*n.c.: not considered*.

**Table 2:** Studied long-term storage conditions.

**Storage condition**	**T (°C)**	**RH _**target**_ (%)**	**RH _**measured**_ (%)^†^**	**Indicative equivalent storage/transportation conditions**
S0	20	50	55 ± 3	Initial condition for filling and sealing
S1	50	10	7 ± 3	E.g., deep-sea container shipping
S2	35	75	80 ± 3	E.g., storage tropical region
S3	35	20	12 ± 3	E.g., transport condition corresponding to the air 50%RH at 20°C and heating up to 35°C
S4	35	20	12 ± 3	Following S3 with a sleeve around the bottle shown in the Figure 1e

† *RH average value measured during the experiment*.

Mass transfer was monitored regularly by weighing the whole bottles (bottle, content and sealing) with an electronic precision balance (±0.001 g, PE160, Mettler Toledo, USA). At regular times, some bottles were sacrificed (opened) randomly to enable ethanol content determinations. The contents of the tested bottles were transferred to glass measurement tubes. Alcohol strength by volume of each sample was subsequently determined at three different depths in the tube using an automatic densimeter (model DMA 5000, Anton Paar GmbH, Austria; accuracy of 10^−5^ % with pure water-ethanol mixtures) equipped with an accurate temperature controller and an autosampler. Ultrapure water was used as reference.

### Databases and Properties

The accuracy of the whole calculations is highly sensitive to the quality of thermodynamic data over the range of temperatures and *abv* values of interest.

*Vapor saturation pressures of pure components*. Saturation pressures of water and ethanol were approximated with a good accuracy via the modified Goff and Gratch equation (see Equation 17) (Goff and And Gratch, 1946; Goff, 1957) and with the Antoine equation (see Equation 18) (Ddbst, 2018) for water and ethanol, respectively.

With pressures in Pa and temperatures in K, $p_{w,sat}^{\left(T\right)}$reads:

(17) $$\begin{array}{l} \begin{array}{c} \underset{10}{\log}p_{w,sat}^{\left(T\right)}=-7.90298\left(\frac{T_{w,sat}^{}}{T}-1\right)+5.02808\underset{10}{\log}\frac{T_{w,sat}^{}}{T}\\ -1.3816\cdot 10^{-7}\left(10^{11.344\left(1-\frac{T}{T_{w,sat}^{}}\right)}-1\right)+8.1328\cdot 10^{-3}\\ \left(10^{3.49149*\left(1-\frac{T_{w,sat}^{}}{T}\right)}-1\right)+\underset{10}{\log}P_{\text{std}} \end{array} \end{array}$$

with all temperatures in Kelvin and the water saturation temperature $T_{w,sat}^{}=373.15\;K$ chosen at the standard pressure *P*_*std*_ = 101324.6 Pa.

Similarly, $p_{e,sat}^{\left(T\right)}$ reads:

(18) $$\begin{array}{l} \underset{10}{\log}p_{e,sat}^{\left(T\right)}=\text{2}.12490+A-\frac{B}{C+T} \end{array}$$

with *A* = 8.20417, *B* = 1642.89, *C* = 230.3 for −57 ≤ *T* ≤ 80°*C*.

*Density of water-ethanol mixtures*. Equation (12) requires accurate determinations of$\rho_{w+e}^{\left(T,abv\right)}$. Values in vacuum have been initially tabulated by OIML (1975) ranging from −20°C up to 40°C, with factual errors discussed in Chanson (2015) to include the revised formula proposed by Bettin and Spieweck (1990). The upper limit of 40°C was imposed by the decrease of the boiling point of ethanol at low pressures. In this study, an extensive database was compiled by assembling the OIML data and by extending them with the predictions of the commercial software AlcoDens (version 3.3, Katmar Software, USA) above 40°C. The slight compressibility of the mixture at atmospheric pressure was based on the compressibility values was corrected from the values reported by the US National Bureau of Standards [see Table 6 of CFR (2006) originated from Osborne et al. (1913)].

*Activity coefficients of water and ethanol in hydroalcoholic mixtures*. Vapor-liquid equilibria of hydroalcoholic mixtures were calculated from the UNIFAC contribution method (Wittig et al., 2003).

*Binary diffusion and sorption isotherms of water and ethanol in PET*. Amorphous PET exposed to water (Burgess et al., 2014a) and ethanol (Chandra and Koros, 2009) above 35°C exhibits complex behaviors on long-time scales. Polymer and relaxation and densification are associated to non-local effects and because they are slower than normal diffusion in thick materials, they were neglected in the current study as well as the possible cooperative interactions between water and ethanol. As reported by Burgess et al. (2014a) and Dubelley et al. (2017), $D_{w}^{\left(T\right)}$ was considered independent of the amount of absorbed water and uniform across the wall thickness and along the contour of the bottle. The effect of orientation during blowing was not considered in agreement with observations of Swaroop and Gordon (1980). The sorption isotherm of water was based on a conservative, but realistic, approximation $f_{w,P}^{T_{0}}\left(RH\right)=\frac{RH}{100}C_{w,sat}^{\left(T\right)}$, where is the saturation concentration $C_{w,sat}^{\left(T\right)}$ of PET. A similar approximation was applied for ethanol based on the experimental values of $C_{e,sat}^{\left(T\right)}$in soaking experiments. Binary diffusion and sorption properties for all tested conditions are summarized in Table 3 along with their strategy of estimation. Since all properties were inferred from literature or independent measurements, the results were compared to experimental measurements without any adjustment.

**Table 3:** Thermodynamic and transport properties of water and ethanol in PET.

**Diffusivities**	**Values**
$D_{w}^{T=35{}^{\circ}C}$	^a^1.5 × 10^−12^ m^2^·s^−1^
$D_{w}^{T=50{}^{\circ}C}$	^b^2.6 × 10^−12^ m^2^·s^−1^
$D_{e}^{T=35{}^{\circ}C}$ when abv = 0.15	^c^5.5 × 10^−16^ m^2^·s^−1^
$D_{e}^{T=35{}^{\circ}C}$ when abv = 0.4	^c^6.3 × 10^−16^ m^2^·s^−1^
$D_{e}^{T=35{}^{\circ}C}$ when abv = 0.7	^c^8.9 × 10^−16^ m^2^·s^−1^
$D_{e}^{T=50{}^{\circ}C}$when abv = 0.7	^d^2.4 × 10^−15^ m^2^·s^−1^
**Saturated concentration**	**Values**
$C_{w,sat}^{T=35{}^{\circ}C}$	^e^0.0118 kg· kg^−1^ dry PET
$C_{w,sat}^{T=50{}^{\circ}C}$	^f^0.0107 kg· kg^−1^ dry PET
$C_{e,sat}^{T=35{}^{\circ}C}$	^g^0.0208 kg· kg^−1^ dry PET
$C_{e,sat}^{T=50{}^{\circ}C}$	^h^0.0224 kg· kg^−1^ dry PET

a *From the Supplemental Information of Burgess et al. (2014a)*.

b *By assuming an activation energy of 49 ± 5 kJ/mol extracted from Table 1 between 23 and 50°C, and averaged over the range of RH between 10 and 90% of Dubelley et al. (2017)*.

c *From Figures 6 and 12 with the conversion between abv and p/po from Figure 2 of Chandra and Koros (2009)*.

d *From D_e_(35°C, abv = 0.7) calculated with Arrhenius activation by T an apparent activation energy 54 kJ/mol*.

e *From Figure 3 of Burgess et al. (2014b)*.

f *From Table 1 of Launay et al. (1999)*.

g *From Table 3 of Chandra and Koros (2009)*.

h *From inhouse vapor sorption experiments with the help of a gravimetric microbalance (model: DVS Resolution, Surface Measurement Systems Ltd.)*.

### Simulation Optimization of Bottle Shapes and Geometry

Simulation optimization refers to the optimization of the shape, wall thicknesses according to a set of constraints, including internal capacity, one or several shelf-life criteria. The problem itself is slightly different from algebraic model-based mathematical programming as it couples physical modeling with several optimization loops and calculations of constraints, which may be not algebraic (i.e., not related to algebraic operations, namely, addition, subtraction, multiplication and division). The objective function defining the quantity to maximize or minimize (e.g., the weight of a packaging unit, shelf-life) and constraints (e.g., capacity, shape ratios) need to be calculated iteratively from independent numeric simulations. According to the considered problem, the number of objectives to consider simultaneously can be reduced by treating some objectives by constraints.

**Figure 2:**
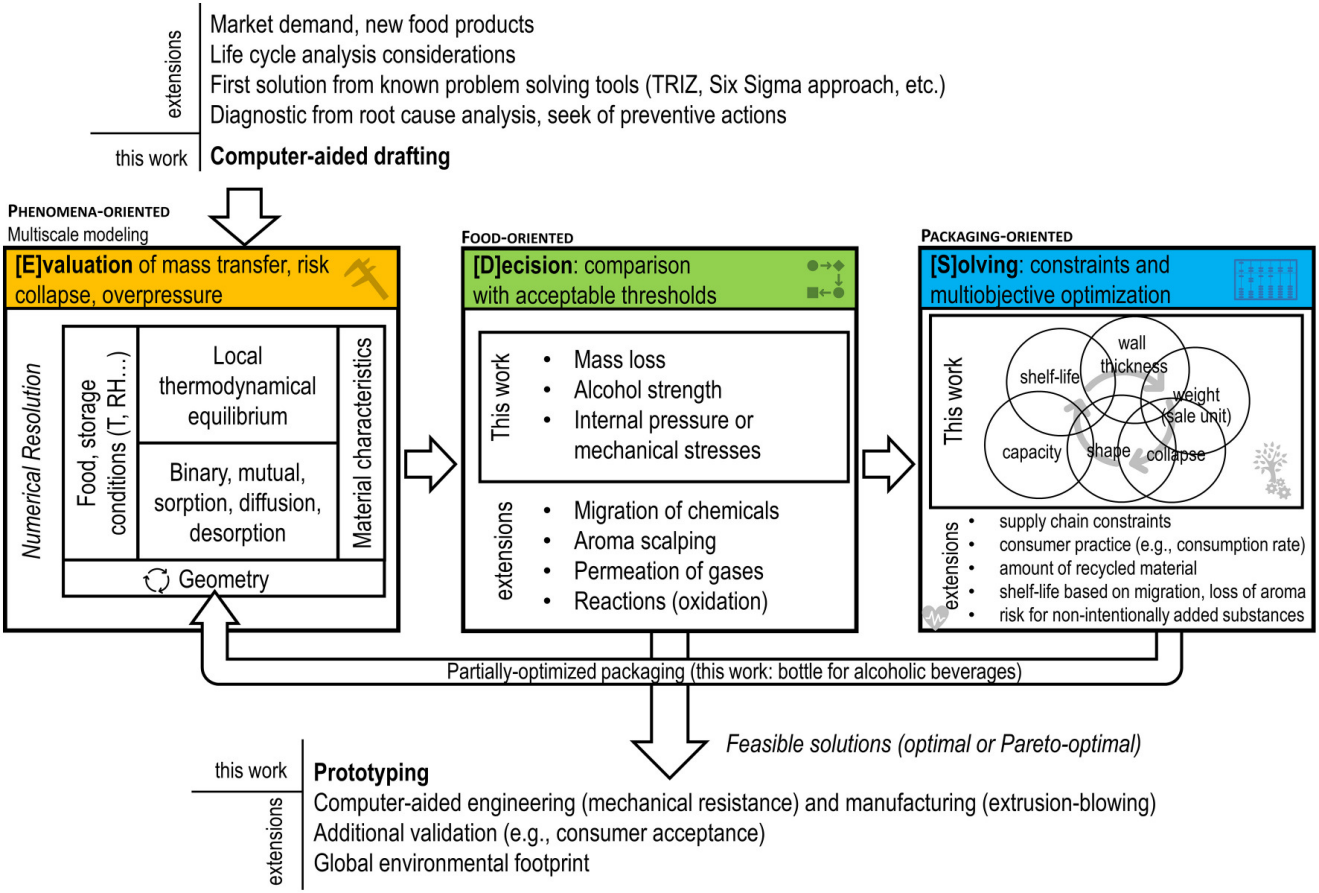
Overview of the simulation-optimization framework and its integration with product-design tools used commonly by industry. Extensions are applications, which are covered by the proposed approach but not applied in the presented case-study.

We propose a global framework to resolve the coupling between 3D geometries and mutual mass transfer problems (sorption, diffusion, desorption) mathematically. At the expense of adding more solutes (from the food, the packaging or the surrounding), the framework is enough flexible to manage one or multiple linked optimization problems associated to mass transfer phenomena and thermodynamics: shelf-life maximization, packaging weight reduction, minimization of the risk of collapse, minimization of migration issues (for one or several substances). The degrees of freedom are the geometry itself and the shape constraints defined by the designer. As a result, an algorithm executed by a computer can explore iteratively several generations of new designs and provide a much smaller number of feasible solutions meeting goals and adhering closely to several constraints fixed by the end-user, current regulations. The simulation-optimization strategy implemented in this work is illustrated in Figure 2. The global task is split into three independent tasks, denoted [E][D][S]: [E]valuation of mass transfer, [D]ecision, respectively, to targets and [S]olving coupled constraints and goals. Taken individually, the method to resolve each task has been explored and justified by their respective field: chemical engineering, risk, and impact assessment, applied mathematics. As an illustration, mass transfer controlling shelf-life and the safety of food contact materials have been detailed in section two, as well as by Vitrac and Hayert (2005) and by Nguyen et al. (2013). Acceptable thresholds are defined either by regulations or by technological considerations (see an overview in the seminal books of Piringer and Baner, 2008; Robertson, 2016). [S]olving the optimization problem, where some objective functions or constraints are nonlinear, can be carried out by extending well-known techniques used in convex optimization and linear programming techniques, such as the simplex method (Strongin and Sergeyev, 2000; Borwein and Lewis, 2006). Once packaged in a single software or library, the whole [E][D][S] loop is intended be combined with tools commonly used by industry, such as Life Cycle Assessment (ISO, 2006), the data-driven Six Sigma approach (Pyzdek, 2003), the TRIZ method or “the theory of inventive problem solving” (Ilevbare et al., 2013). A notable feature of the approach is to use 3D representations, making it virtually interoperable with computer-aided design and drafting tools (solid and surface modelers), engineering and finite element tools (mechanical resistance of bottles), manufacturing tools (e.g., extrusion blowing of preforms).

**Figure 3:**
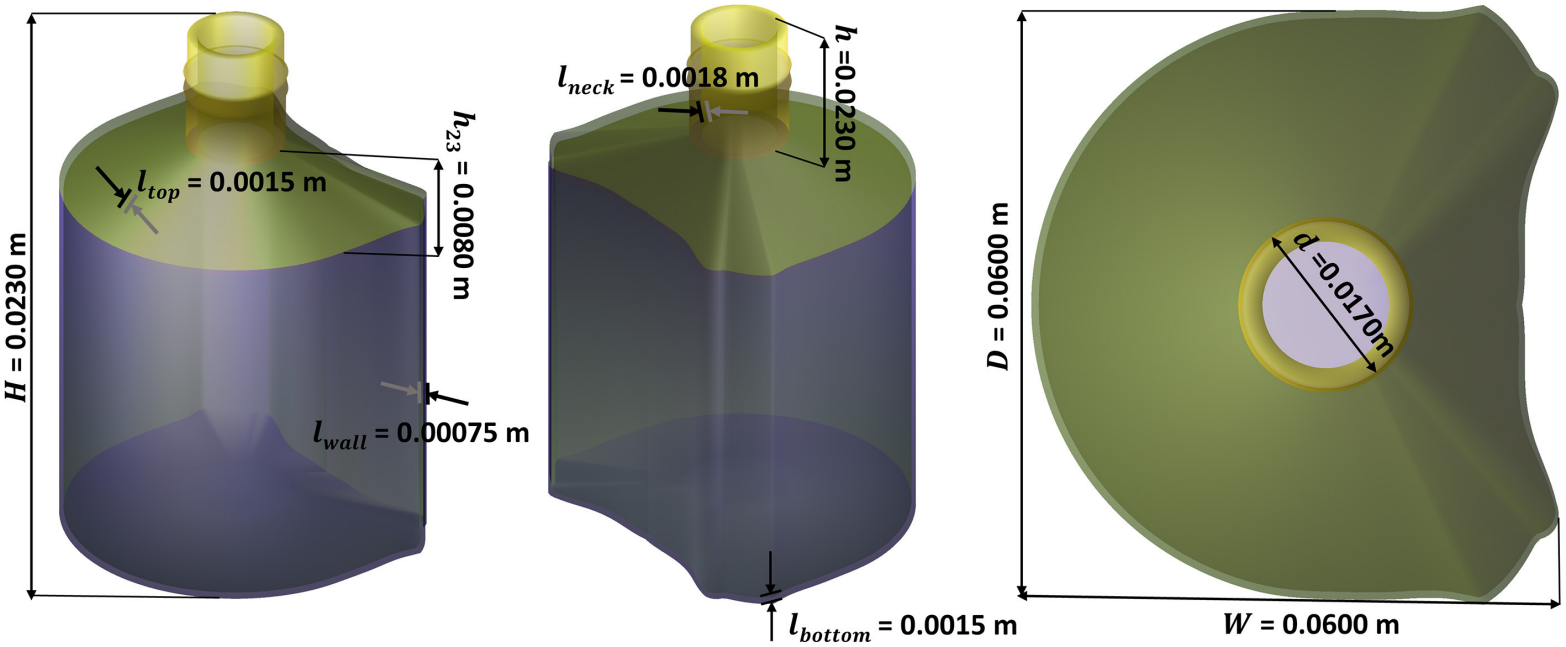
Example of initial bottle design to be optimized.

Nonlinear and non-convex problems such as shelf-life and weight optimization problems remain intrinsically more difficult to [S]olve. The optimal solution may be not unique, it may occur at an interior point of the feasible region, at its boundary or at one the extreme points of the feasible region. Integrating uncertainty inherent to any [E]valuation step complicates the definition of a robust termination condition. The best solution lies within a tolerance from the last best optimum and at an acceptable distance of critical constraints to be considered “safe” or “compliant” ([Decision step]). In this work, the internal domain is mapped with a regular grid to offer to enable a sensitivity analysis close to constraints. An instance of the [S]olving step is detailed for a packaging design problem with a prescribed capacity (150 mL). The shape of the bottle is homothetic to the 3D shape presented in Figure 3 (non-optimized) while matching exactly an internal volume of 160 mL for a nominal capacity of *V*_*beverage*_ = 150 mL and a shelf-life of at least 180 days at 25°C for aa vodka-type beverage (*abv*_0_ = 0.4).

Based on the geometry parameters defined in Figure 3, the weight optimization problem was split into a two-step optimization process: i) setting macroscopic dimensions *H, W, D, d, h* to match the prescribed internal volume and ii) optimizing *l*_*wall*_, *l*_*bottom*_, *l*_*top*_, *l*_*neck*_ to adjust the barrier properties of the bottle. Without a loss of generality, the general problem is solved using a multiresolution method [see Amaran et al. (2014) for a review of applicable algorithms]. At the lowest level, all parameters in the group are frozen except one. The elementary problem with shelf-life and capacity constraints reads hence:

(19) $$\begin{array}{l} \begin{array}{c} l_{wall}\left(X\right)=\arg\min\left[\begin{array}{c} mass_{bottle}\left(X,l_{wall}\right)\\ \text{with }shelf\text{-}life\left(l_{wall},X,abv_{0},V_{beverage},T\right)\\ \geq 180\text{ days} \end{array}\right]\\ \text{where }X={\left[V_{bottle},W,D,d,h,l_{top},l_{bottom},l_{neck}\right]}^{\prime }\text{and }V_{head}\\ =V_{bottle}-V_{beverage}\geq 0\left(19\right) \end{array} \end{array}$$

where the shelf-life function is defined as:

(20) $$\begin{array}{l} \begin{array}{c} shelf\text{-}life\left(l_{wall},X,abv_{0},V_{beverage},T\right)=\\ \min\left[\begin{array}{c} \arg\min\left(|abv\left(t,l_{wall},X,abv_{0},V_{beverage},T\right)|\geq 0.003\right),\\ \arg\min\left(m_{beverage}\left(t,l_{wall},X,abv_{0},V_{beverage},T\right)\geq 1.5\%\right) \end{array}\right] \end{array} \end{array}$$

and where the height of the bottle is found by adding the complementary constraint:

(21) $$\begin{array}{l} H(V_{beverage},l_{wall})=\text{arg min }[|V_{\text{internal}}(H,l_{wall},V_{bottle})\\ -V_{beverage}|] \end{array}$$

As a result, the internal loop manages implicitly design and product constraints in a conventional simulation framework. All input parameters are set conventionally except *H* and *l*_*wall*_, which are optimized by resolving successively the capacity and the shelf-life problem via a golden-section search method (see section 10.1 in Press, 1992). Additional degrees of freedom controlling the shape of the bottle are introduced at a higher tier by resolving again the elementary problem. The current implementation is sufficiently efficient to screen hundreds or thousands of geometries/designs under constraints. The global optimization when *W* and *D* are released has been explored on a 30 × 30 grid (i.e., 900 bottle geometries were optimized).

## Results and Discussion

The problem of mass transfer between an alcoholic beverage and the surrounding ambient is described via quantities, which are causally related to the shelf-life of the product: mass loss (Δ*m*) and the residual alcoholic strength (*abv*). Mass loss may be, however, ambiguous whether it is defined as the net variation of the amount of the consumable product (i.e., legal definition) or as the net variation of the beverage mass plus its packaging (i.e., permeation definition). The second one was used in this study. The difference between the two definitions is related to the amounts of water and ethanol present in the walls. A correction by subtracting the initial mass of the container was avoided as the walls (bottles were stored at 50% RH prior experiments) were also containing water. With this respect, the parameter *abv* is only associated to the content of the package (destructive measurement), but its variation may evidence ethanol loss (by sorption or permeation), water loss (by sorption or permeation) or more likely a combination of both. Detailed simulations were used to reconstruct the behavior of an average container exposed to controlled conditions. The reconstruction for individual bottles was out of each as they depended on factors out of our control and which are listed in decreasing order of importance: leaks from the lid, volume filling errors, ethanol evaporation before sealing, distribution of PET material in the bottle (thickness variation between bottles), the mass of the bottle.

### Driving Forces Controlling Shelf-Life

**Figure 4:**
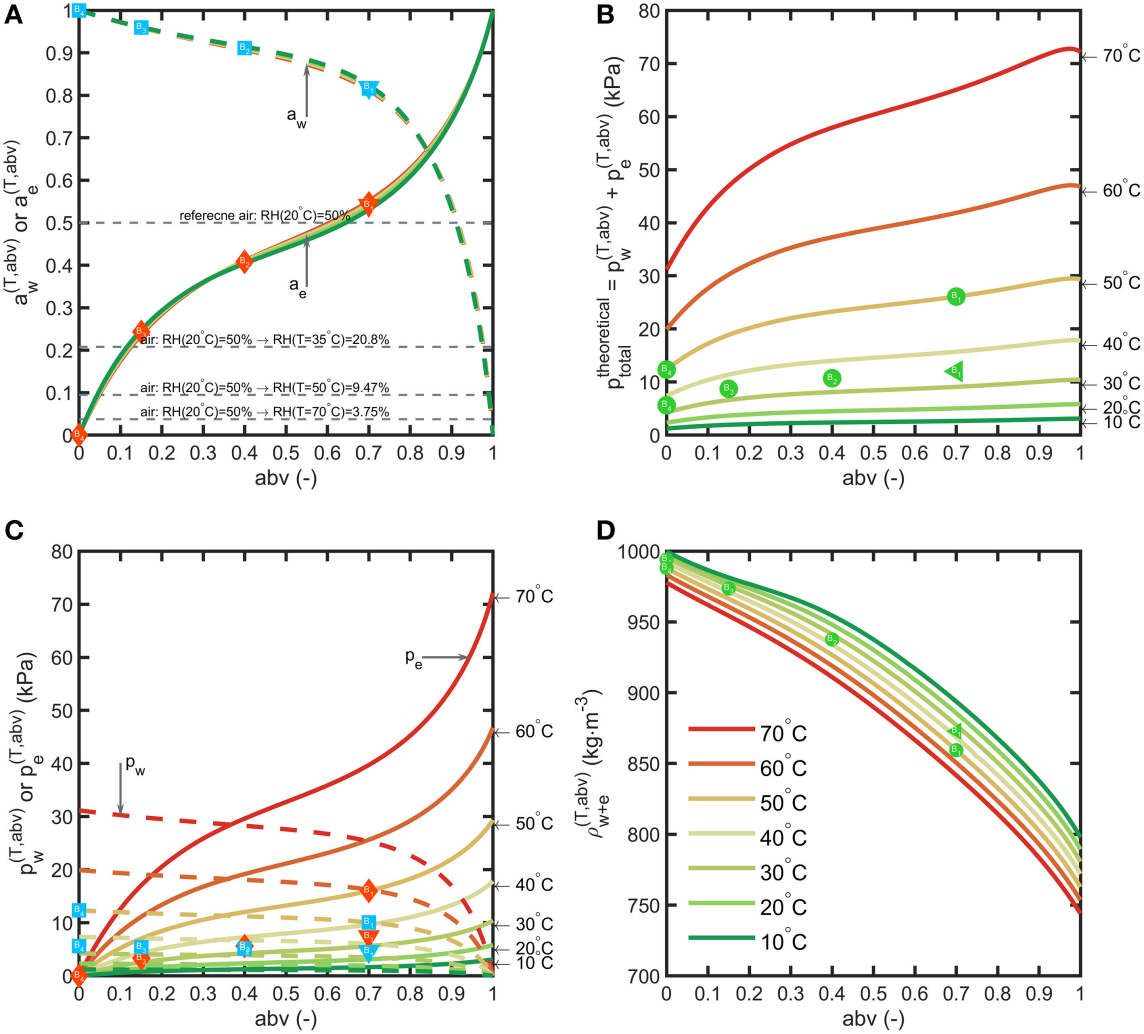
Binary properties of water-ethanol mixtures: **(A)** activities, **(B)** partial pressures, **(C)** total pressure in vacuum, **(D)** liquid density. The symbols depict the tested beverages and storage conditions. Horizontal lines in **(A)** show the variation of RH when air temperature is increased from 20°C up to 70°C.

By noting that PET is not a porous material, water and ethanol mass transfer across bottle walls occur prior dissolution in the polymer and obey to the general sorption-diffusion-desorption model. The main driving forces are the partial pressure differences between the beverage and the surrounding. The variations of binary properties of water-ethanol mixtures with *abv* between 10 and 70°C: mixture density $\rho_{w+e}^{\left(T,abv\right)}$, activities ${\left\{a_{i}^{\left(T,abv\right)}\right\}}_{i=e,w}$, partial pressures${\left\{p_{i}^{\left(T,abv\right)}\right\}}_{i=e,w}$and the theoretical pressure of the headspace at constant volume (no air) $p_{total}^{theoretical}=p_{w}^{\left(T,abv\right)}+p_{e}^{\left(T,abv\right)}$ in equilibrium with the mixture are presented in Figure 4.

By neglecting the amounts transferred to the headspace, binary equilibrium data offer a first approximation of the driving forces based on the alcohol strength (*abv*) of the considered beverages and the conditions of storage. Activities of water and ethanol do not evolve significantly with temperature. Water activities at 35°C of typical alcoholic beverages correspond to 0.977 (*abv* = 0.08), 0.960 (*abv* = 0.15), 0.911 (*abv* = 0.4) and 0.819 (*abv* = 0.7) as lower limits for beer, wine, vodka, rum, respectively. In non-tropical regions (for RH < 0.8), alcoholic beverages stored in plastic bottles lose water and therefore weight. Ethanol activities are comparatively much lower 0.153 (*abv* = 0.08), 0.243 (*abv* = 0.15), 0.408 (*abv* = 0.4) and 0.541 (*abv* = 0.7). The effective driving forces $a_{w}^{\left(abv\right)}-\frac{RH\left(T\right)}{100}$ and $a_{e}^{\left(T,abv\right)}-0$ across bottle walls tend to be of the same magnitude order in dry conditions. Temperature affects differently the partial pressure of water and ethanol. Increasing the temperature in a closed storage room will cause a dramatic decrease in *RH*(*T*) and therefore of water transfer. In our experiments, condition S1 is the most extreme and corresponds to conditions met in container transport. The cumulated pressures $p_{total}^{theoretical}$ offer a lower bound for the total pressure reached in the headspace under vacuum (no air). It can reach up to 25 kPa for liquors at 50°C (point B1(abv = 0.7, $p_{total}^{theoretical}$ = 25 kPa) in Figure 4B).

**Figure 5:**
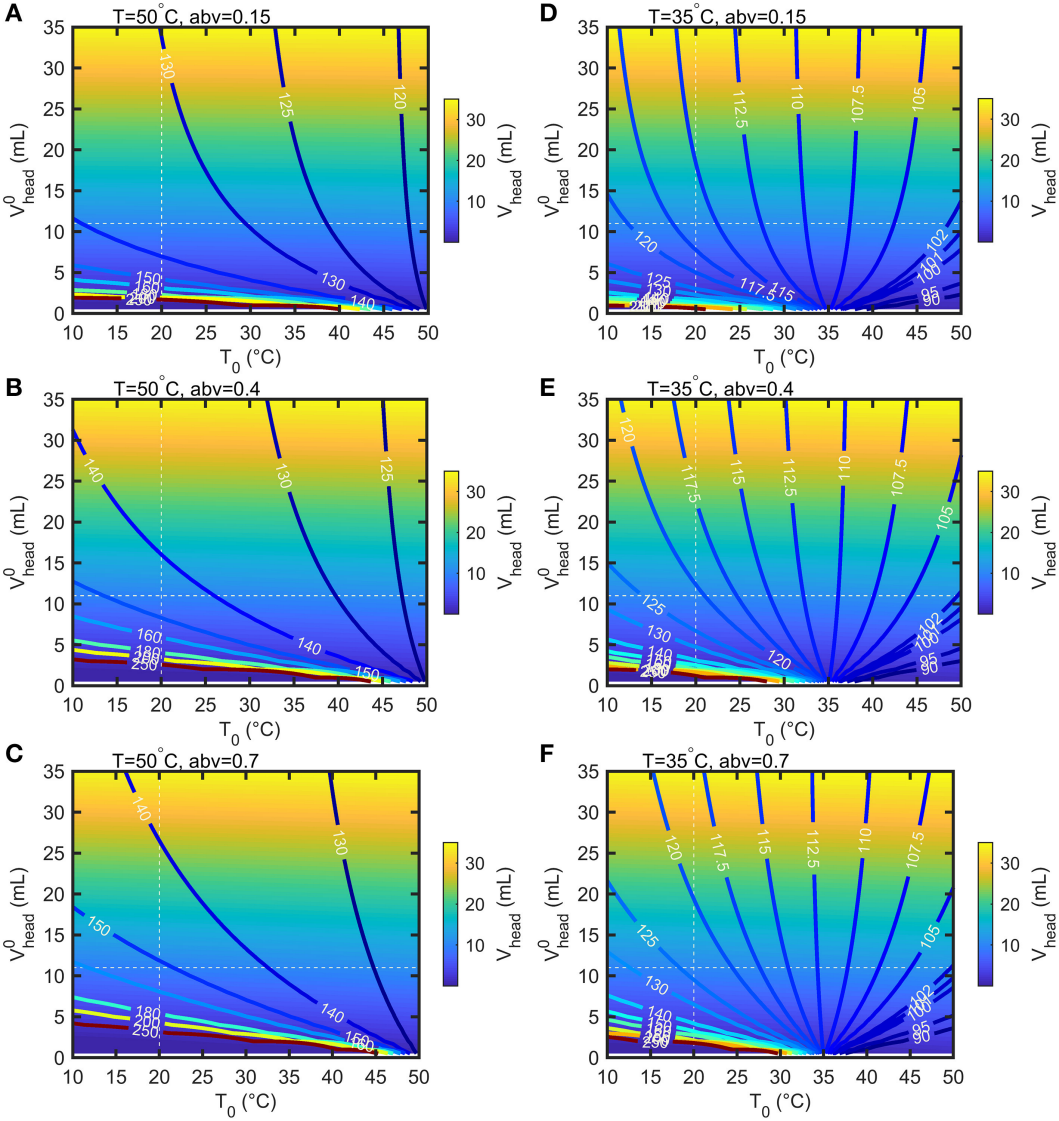
Calculated iso-headspace pressures (*P*_*head*_) and iso-headspace volume (*V*_*head*_) at equilibrium in the bottle depicted in Figure 1 according to the headspace volume ($V_{head}^{0}$) and temperature (*T*_0_) at filling time. The results are presented for two storage temperature 50°C **(A–C)** and 35°C **(D–F)** three abv values (0,15, 0.4, 0.7).

Bottles are filled with air at atmospheric pressure and at a temperature lower than the storage temperature. These conditions will provoke significant evaporation of ethanol and in a less extent of water, as well as thermal expansion of the mixture (see Figures 4B,C). According to the volume of headspace, *V*_*head*_, the pressure difference between the headspace, *P*_*head*_, and the surrounding, *P*_*atm*_ = 100 kPa, can be much greater than $p_{total}^{theoretical}$. Calculations were carried out for the 55 mL bottles depicted in Figure 1 with a headspace volume varying from 0.5 to 35 mL and by assuming that the walls of the bottles are rigid. When the filling temperature *T*_0_ is equal to the storage temperature *T*, the internal overpressure, *P*_*head*_−*P*_*atm*_, is commensurable to $p_{total}^{theoretical}$ and independent of *V*_*head*_. Using storage temperatures greater than *T*_0_ provokes strong overpressures strongly dependent on *V*_*head*_ and on *abv*. In our experimental conditions *V*_*head*_≈11 mL and $T_{0}=20{}^{\circ}C$, *P*_*head*_ reaches 135, 145, 152 kPa at *T* = 50°*C* and 116, 121, 123 kPa at *T* = 35°*C* for *abv* = 0.15, 0.4, 0.7 shown in Figure 5, respectively. *V*_*head*_decreases from 11 mL down to 10.43, 9.99, 9.67 mL at *T* = 50°*C* and 10.76, 10.51, 10.36 mL at *T* = 35°*C* for *abv* = 0.15, 0.4, 0.7, respectively. Dividing *V*_*head*_by 2 and 4 in the worst-case conditions (*T* = 50°*C*, *abv* = 0.7) increases the overpressure by 17.4 and 91%. Negative pressures (below *P*_*atm*_) associated with important risk of collapse occurs in presence of headspace volumes lower than 5 mL and filling temperatures 10°C above the storage temperature ($T_{0}>T+10{}^{\circ}C$).

A significant total pressure difference across the lid existed for all tested storage conditions and beverages including when the bottles were filled with pure water. For *abv* > 0.4, the internal pressure was estimated 86 % higher in the presence of ethanol than without and made the risk of leaking through the more likely. Leaking was detected by a rapid mass loss during the week of storage. The number of leaking bottles reached 48% at 50°C and 23% at 35°C for *abv*≥0.4 (see Table 1). For low*abv*values (0.15), the number of leaking bottles dropped down to 9.4% and was reduced to zero in the case of pure water.

### Coupled Water-Ethanol Mass Transfer From 50 mL Bottles

**Figure 6:**
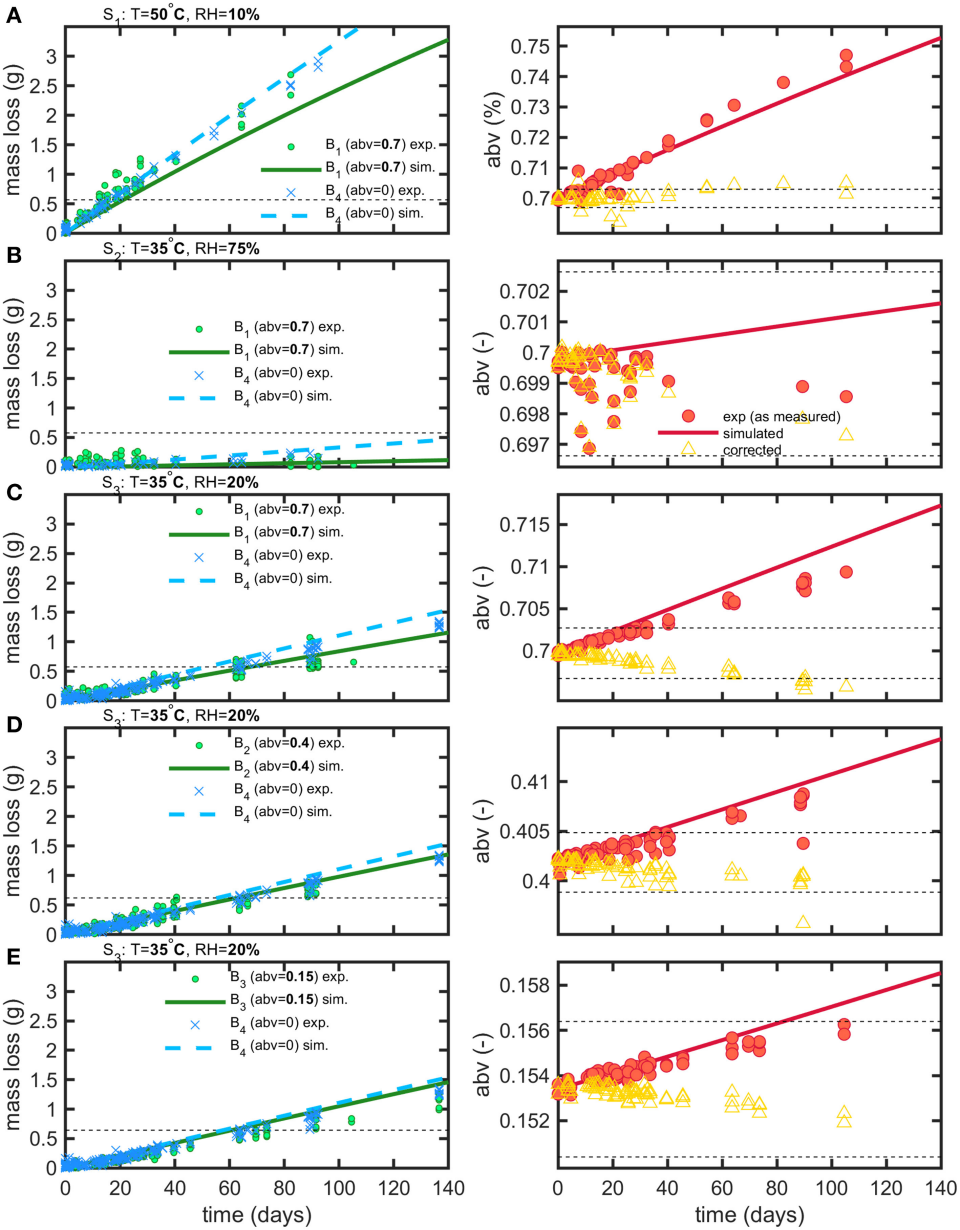
Comparison of experimental (exp) and simulated (sim) mass loss and abv variations for storage conditions S1 **(A)**, S2 **(B)**, S3 **(C–E)** and beverages B1 **(A–C)**, B2 **(D)**, B3 **(E)**, B4 **(A–E)**. The dashed lines plot the thresholds used to calculate shelf-life (see text). Empty symbols (triangles) represent the theoretical variation of abv when concentration effects due to water permeation are corrected.

**Figure 7:**
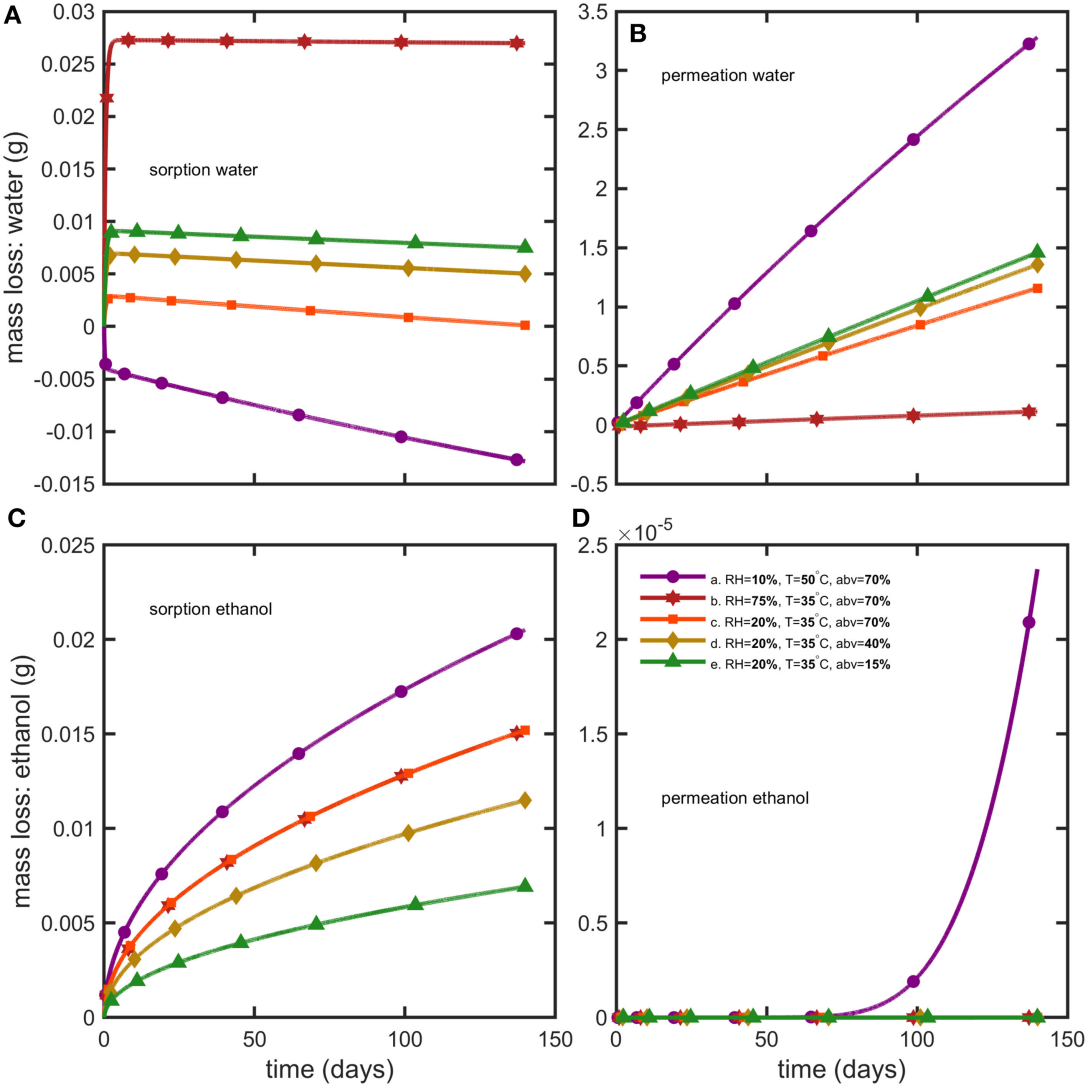
Reconstruction of mass losses by sorption and permeation for **(A,B)** water and **(C,D)** ethanol.

After discarding leaking bottles, 1889 mass measurements and 286 *abv* determinations were collected on 358 bottles for all tested conditions (see Table 1). Bottle weights were monitored during a period of up to 5 months with bottles regularly sacrificed to allow *abv* determinations. The same experimental conditions were simulated using an axisymmetric bottle geometry averaged over ten bottles (see Figure 1) and using an averaged filling volume for each tested condition. Temperature and relative humidities introduced in boundary layers were based on the average of log values. Experimental and simulated mass losses and *abv* values are reported in Figure 6 with the individual fluxes of water and ethanol reconstructed by simulation detailed in Figure 7. Experimental losses were also standardized to the same filling weight to remove the initial dispersion of data. To facilitate the interpretation of coupled water-ethanol mass transfer, the permeation curve of water alone is shown as reference for all tested conditions. The variations of *abv* corrected from water loss are also presented by assuming that the permeation of water was proportional to the applied driving forces.

Weight differences between bottles filled with water and hydroalcoholic mixtures are little and controlled by the differences in water activities, $a_{w}^{\left(abv\right)}-\frac{RH}{100}$, on both sides of bottle walls. Though water losses dominated in alcoholic beverages, the shelf-life was determined mainly by the variation of the *abv* criterion in hydroalcoholic mixtures. The maximum storages times fulfilling weight loss and *abv* criteria are summarized in Table 4 for the strictest conditions associated to containers larger than 1L and extrapolated down to containers of 50 mL. The rapid permeation of water trough bottle walls caused a concomitant fast increase in ethanol strength (*abv*) in conditions S1 and S3 (see Figures 6A,C,D). Weight losses exceed faster limits only for mixtures with low *abv* values (Figure 6E) or when the difference of water activities was low (2b). For both observed criteria (*abv* and weight loss), a steady regime was reached within 3 to 6 days. Simulations enabled to reconstruct all aspects of mass transfer for all tested conditions and provided a mechanistic interpretation of coupled mass transfer. Figure 7 decomposes mass losses for water and ethanol according to sorption and permeation. Contact times were much longer than permeation lag-times (Figure 7B) and justified the apparent steady state of weight losses and *abv* variations. The water content in bottle walls evolved in a non-linear manner with desorption dominating in very dry conditions (condition S1). At high (S2) and intermediate (S3) relative humidities, the rapid water sorption is followed by a slow drying when the surrounding *RH*during storage is lower than the equilibration *RH*before the experimentation starts (Figure 7A). Ethanol diffuses slowly through PET without reaching a steady state. Lag-times beyond which permeation of ethanol may reach a steady state are evaluated ca. 1.4 and 3.6 years, at 35 and 50°C, respectively.

**Table 4:** Comparison of shelf-lives extrapolated from experiments and calculated from simulations.

**Storage condition**	**Beverage**	**Time to reach 1.5% m/m mass loss (days) t_*m*_**		**Time to reach ± 0.3% variation of abv (days) t_*a*_**		Theoretical shelf-life for 50 mL bottles^*^ **(days)** $\text{min }\left(\frac{9}{1.5}t_{m},t_{a}\right)$	
		**^**exp.**^**	**^**sim.**^**	**^**exp.**^**	**^**sim.**^**	**^**exp.**^**	**^**sim.**^**
S1	B1	18 (16.20)^a^	21.1	8.5 (8,9)^a^	7.3	8.5	7
S1	B4	17 ± 1.5^b^	19.2	–	–	102	115
S2	B1	>140	>730	>140	225	>140	224
S2	B4	>140	197		–	>140	1182
S3	B1	64 (63.65)^a^	67.1	31 (29.33)^a^	22.9	31	22
S3	B4	64 (63.65)^a^	58.6	–	–	384	351
S3	B2	54 ± 5^b^	62.3	38 (36.40)^a^	33.5	38	33
S3	B4	66.5 (64.69)^a^	58.6	–	–	399	351
S3	B3	69 (64.74)^a^	60.7	105	82	105	82
S3	B4	65.5 (64.67)^a^	58.6	–	–	393	351

† see Annex 1 of EU Directive 76/211/EEC (EEC, 1976) (class “B” product with a package capacity over 1 L); ‡see Annex XII of EU regulation 1168/2011/EC (EC, 2011) (applicable for any beverage containing more than 1.2% vol); ^*^shelf-life extrapolated for 50 mL bottles according to EU Directive 72/211/EEC.

*a. shelf-life on average days (min,max); b. shelf-life in days ± 95% confidence interval*.

**Figure 8:**
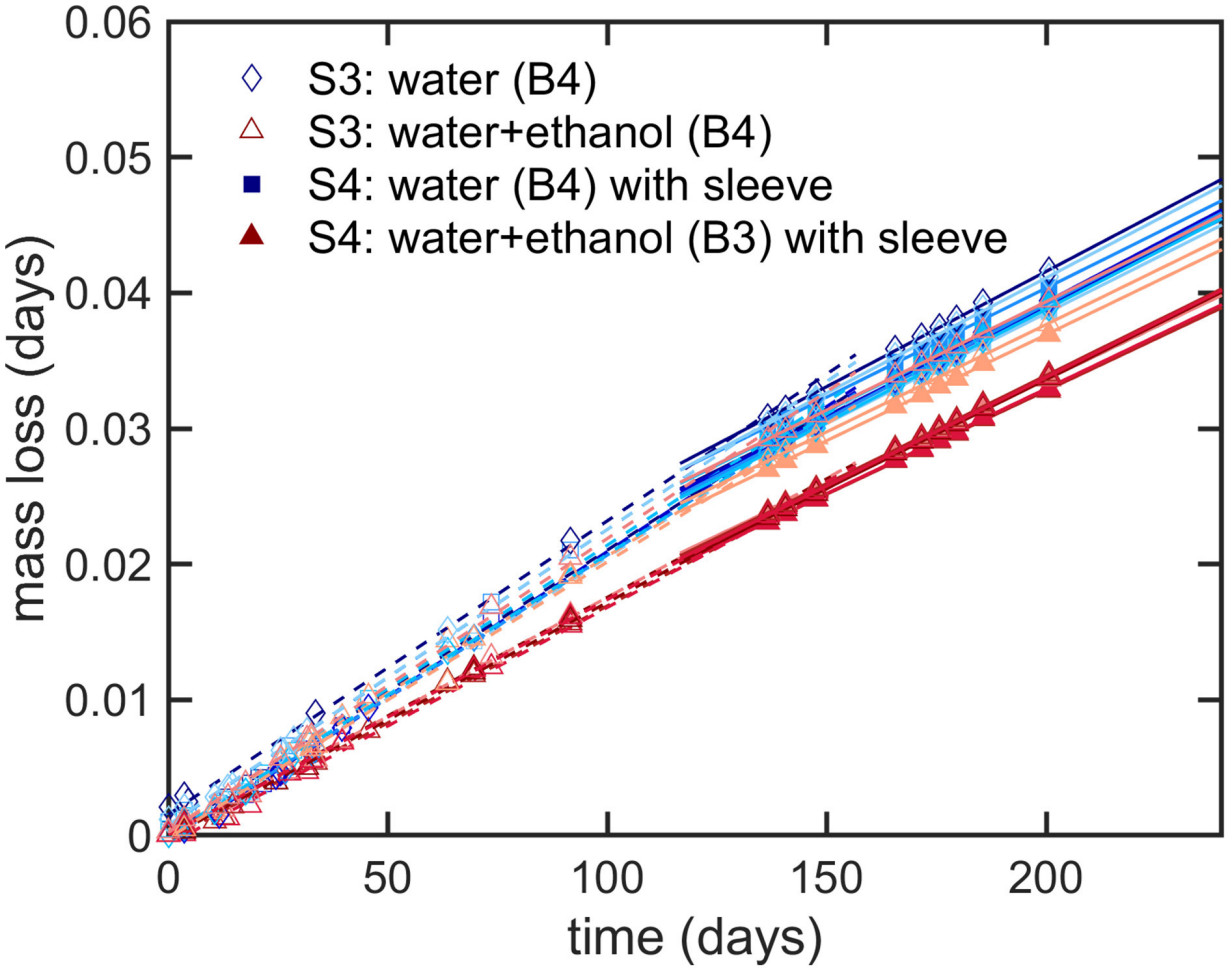
Modifications of mass transfer when a sleeve is added to the bottles containing the beverage B3 and B4 after several months of storage: mass loss during condition S3 (dash lines, one line per considered bottle) and S4 (continuous lines, one line per considered bottle).

The results presented a significant variability between bottles, which could not be reduced experimentally, and which could not be reproduced by simulation. The sealing was identified as the source of major leaks and defects, but the contribution of a weak gas leak, which could be confused with permeation, could not be tested directly along with other sources of variabilities: variable plasticizing of PET and variable distribution of PET material at injection-blowing time. It is worth noticing that possible heterogeneities in hydrodynamic, temperature and humidity conditions around bottles cannot be invoked to explain variabilities, as all climatic chambers were equipped with an efficient air recirculation and renewal. Further insights were brought by adding a shrinking 60 μm biaxially oriented PET film (sleeve) around the thinnest part of the bottle (cylindrical section of the bottle as seen in Figure 1), which was thought to contribute the most to the overall permeation rate. The sleeve was added to bottles of B3 (*abv* = 0.15) and B4 (water only) at the end of storage S3 (see Table 2); it covered ca. 80% of the total surface area. Since ethanol could not significantly cross the ca. Eight hundred micrometer hick bottle walls, the external film reduced mainly the flux of water. Mass losses before and after the addition of the sleeve are analyzed individually for 20 bottles containing ethanol (10) or not (10) in Figure 8. The results were not normalized to highlight the different evolutions of the bottles. Before the addition of the sleeve, the “trajectories” followed by the bottles diverged (different permeation rates). Adding a sleeve inflected all permeation rates in a very similar way and made all permeation kinetics looking very similar with almost parallel slopes. This trend confirmed the prevalence of the body of the bottle and of water permeation on overall mass transfer. Since the thickness of the sleeve was commensurable to the thickness fluctuations in the body region (see Figure 1c), the dispersion of permeation rates between bottles was associated to the slight variations of the distribution of weight after injection-blowing (see similar analyses in Figures 3–6, 10 of Daver et al., 2012; Demirel and Daver, 2012, respectively). The flux reduction followed approximately the rules of serial association of mass transfer resistances: ${\left(l_{wall}^{-1}+l_{sleeve}^{-1}\right)}^{-1}$, with *l*_*wall*_ and *l*_*sleeve*_ the thicknesses of the wall and the sleeve, respectively.

### Optimal Design of Packaging Systems for Liquors

**Figure 9:**
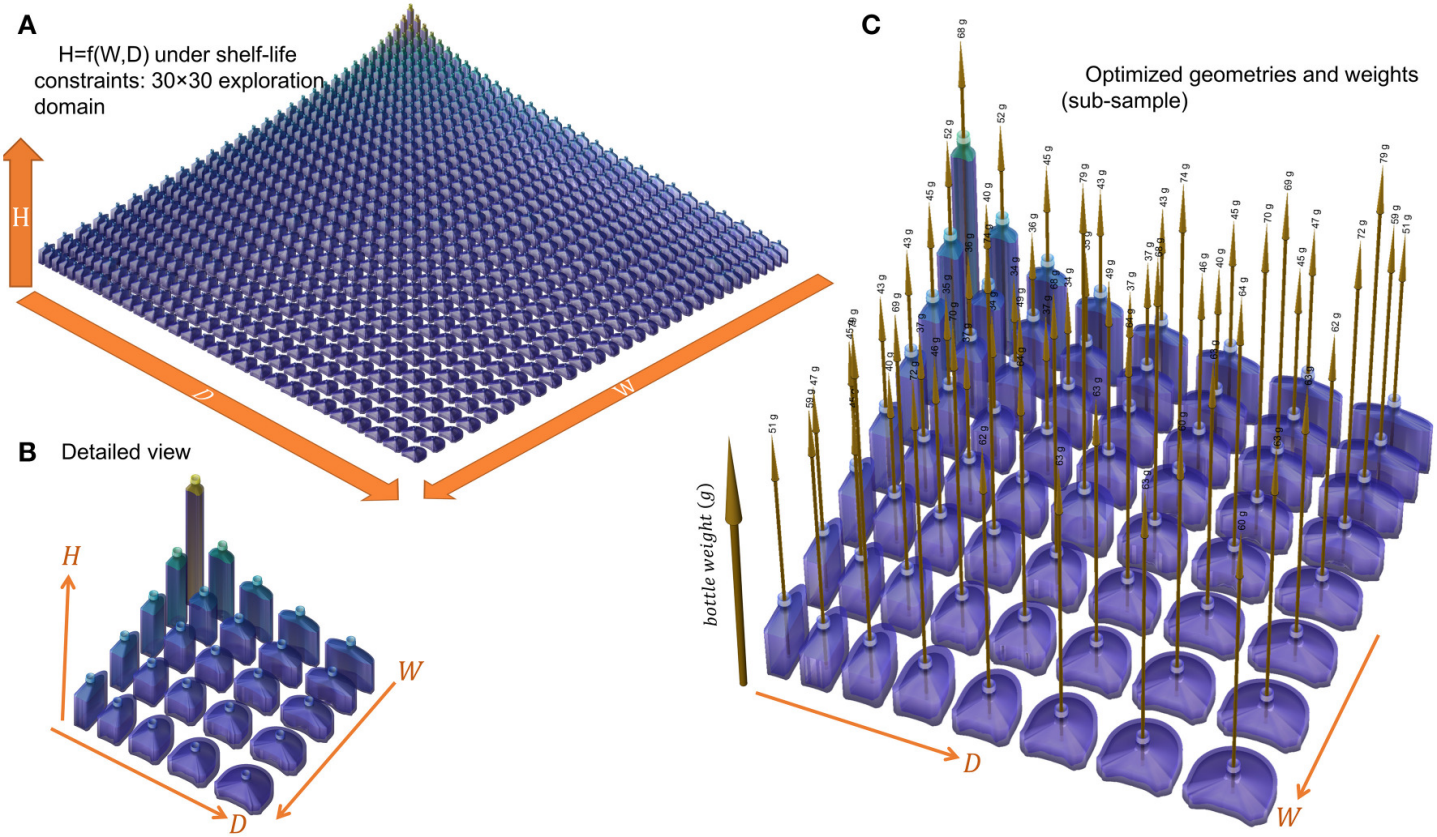
Space of geometries explored and optimized for a 160 mL bottle (capacity 150 mL) containing a vodka-type beverage: **(A)** shapes corresponding to a 30 × 30 combination of *W* and *D*, **(B)** 5 × 5 combination, **(C)** weights of bottles optimized to fulfill a shelf-life of 180 days at 25°C.

**Figure 10:**
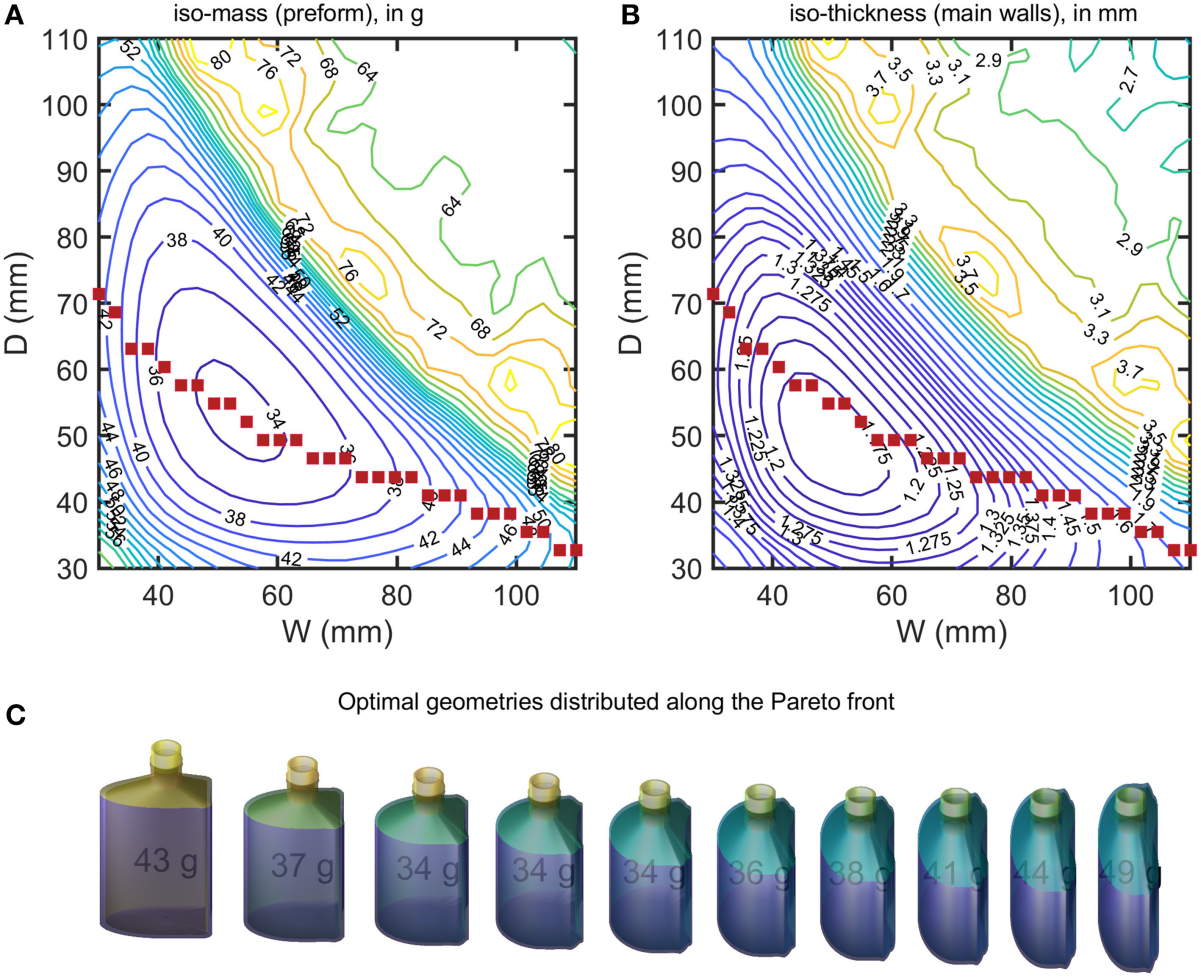
**(A)** iso-weight and **(B)** iso-thickness contours of optimized bottles (minimum shelf-life of 180 days) shown in Figure 9. The filled symbols locate the sampled Pareto front. A selection of designs sampled along the Pareto front (from left to right) is presented in **(C)**.

The design of a new packaging can be seen either as a problem of a relationship between wall thickness and shelf-life or as a more global problem seeking the maximization of shelf-life while minimizing the mass of plastic material. As previously shown on the tested 55 mL bottles, wall thickness and shelf-life are not independent parameters as soon as a steady regime is reached. Under a strict permeation control, doubling shelf-life requires twice the initial thickness and doubles the weight of plastics. Substantial gain can be achieved without damaging weight only if some non-linarites can be introduced in the original engineering problem. They appear spontaneously if the shape and, in particular, the surface-to-volume ratio is also a degree of freedom. Its contribution was investigated by analyzing the effects of parameters *W* and *D* on the weight of bottles matching the design presented in Figure 3. For each value of *W* and *D*, the wall thickness of the main sides was optimized to grant a minimum shelf-life of 6 months for a vodka-type product stored in tempered conditions (25°C at 50% RH). The full explored domain covered a 30 × 30 grid describing a uniform variation of both parameters between 30 and 120 mm. All other properties were recovered by simulation-optimization. It is worth noticing that only *l*_*wall*_ was updated for each bottle. The other constraints were managed by mathematical constraints applied to the bottle geometry. For efficiency and robustness in particular close to edges, all bottles were modeled as cylindrical B-spline surfaces rather than swept surfaces. All integrations (masses, fluxes) were integrated accordingly. The corresponding space of explored shapes is shown in Figure 9 with optimized values summarized in Figure 10. Shapes vary from almost symmetric cylindrical bottles, flask shapes, long tubes, flat bottles. Some shapes were exotic, but they could be examined at no extra cost by calculations.

The optimization procedure generates two families of solutions for the same final capacity 150 mL and shelf-life but with weights varying in a ratio 1:2. Flat geometries associated with large *W* and *D* values led to heavy bottles and large weights. On the contrary square sections with intermediate *W* and *D* values led to very similar weight reductions, while keeping the same shelf-life. The transition between the two regions appears noisy with several maxima. The transition region corresponded to a shift of the shelf-life criterion controlled by *abv* for heavy bottles and by the mass loss for light bottles. For a given *W*or*D*value, a unique bottle minimizes the weight of the PET bottle. The continuous curve connecting all points represents the Pareto front for all the criteria considered: capacity, shelf-life, weight, shape factor. Changing *W*or *D*will at the expense of either the shape of the bottle or of its weight. A selection of bottles along the Pareto front is shown in Figure 10C. The result can be seen as a shape interpolation process from one shape where the curvature is on the smallest side to a final shape where the curvature dominates the largest side. As the bottle is not symmetric, the transformation is not equivalent to a swap of sides. The optimal shape is closer to the cylinder shape and offers as expected the minimum surface-to-volume ratio.

## Conclusions and Perspectives

Minimizing plastic wastes is an urgent priority for our planet. New prototyping tools to explore the consequences (positive or negative) of the packaging design on the shelf-life of sensitive products is a chief priority. It could be though naively than alternative shapes could reduce the weight of packaging dramatically while keeping unchanged shelf-life. For beverages, it is usually not verified as the shapes of commercial bottles tend already to minimize surface-to-volume ratios, at least for those with large capacities. The alternative scenario offers, however, broader perspectives: what is the loss of shelf-life induced by a modification of the format, of the shape or by slimming the walls? The presented computational framework is generic and flexible. It can tackle both design problems, but also analyze the conditions of shelf-life loss.

Integrating simulation and semi-supervised decision making in the ecodesign process is highly desirable. Ecodesign was theorized in the groundbreaking manual of the United Nations Environment Programme (Brezet and Van Hemel, 1997). The strategy is split into seven sub-problems or steps, which could be translated as follows for packaging design: preferring materials with low impacts (S1), minimizing packaging weight (S2), optimizing shape (S3), mitigating transportation and retailing conditions including the risk of overpressure and collapse (S4), updating the packaging format to the food volume consumed (S5), optimizing food shelf-life (S6), optimizing collection, recovery and recycling (S7). Steps S1 and S7 require a global evaluation and should be oriented according to the indices “first impacts” and “end-of-life impacts” common to life cycle analysis. Steps S2 to S5 correspond to a detailed optimization involving the packaging, the food, retailing, and consumer practices or preferences; they can be streamlined to target many optimal or sub-optimal solutions. For instance, the format of the packaging can be optimized by minimizing the amount of packaging waste generated by an average consumer over 1 year (mass of a single packaging unit × number of units consumed in 1 year). The solution should accept shelf-life as a free-parameter bounded by a maximum commercial value and a minimum value combining the retailing time, consumption time and the maximum durability of the product after opening. The different levels of optimization associated to the full problem “less plastics, more shelf-life, exact or better internal capacity, minimum collapse/overpressure, interpolation of the right shape” are resolved iteratively via a three-step scheme, denoted [E][D][S], involving an [E]valuation of mass transfer and thermodynamics; a [D]ecision based on a comparison with acceptable thresholds; and a [S]olving procedure adapted for non-linear problems. Step [E] can be extended to a broad range of phenomena including chemical reactions (oxidation and hydrolysis) and additional mass fluxes (aroma scalping, migration of plastic additives). With this respect, safe-by-design and ecodesign approaches can be treated in parallel without substantial additional cost and by adding rows to Equations (13–16). The only limit is the availability of diffusion and partition coefficients. Step [D] aims at adjusting walls the thickness of the walls, shelf-life or concentrations to fulfill the chosen goals. When many independent phenomena are reducing shelf-life, the latter should be defined as the lowest value imposed by all phenomena considered (e.g., mass loss, scalping, migration, collapse) in the most severe scenario. Finally, the entire geometry can be modified and resolved [S]. Parametric modeling (with many additional geometric constraints) enables to preserve the design intent between dimensions, sections, parts, and assemblies while exploring thousands of combinations. Very few primitives enable to test almost any imaginable bottles in almost real time and to integrate them in the packaging workflow. For example, the approach can resolve what the optimal bottles for given preform are? How to distribute the mass during blow molding.

The detailed case-study demonstrate that very small formats (miniatures) cannot compete with larger PET bottles for storing alcoholic beverages. Similar tolerance on mass loss and alcohol strength by combining two strategies: increasing wall thicknesses and changing their shapes. Additionally, the main stressors have been identified and are associated contrary to intuition to the difference of partial pressure of water across the walls and not the one of ethanol. As the thickening of the wall is beneficial only for water permeation, keeping miniatures in a humid place or even immersed in water can contribute to extending shelf-life without producing additional waste. Beyond brute-force optimization, the [E][D][S] approach can bring a comprehensive description of causes and stimulate the production of alternative designs and industrial practices for alcoholic beverages, but virtually for any packaged food. The gains might be highly substantial. Revised miniatures with triplicate capacity exhibit thus double shelf-life with half-weight. The acceptability of the calculated solutions can be tested in real time in augmented reality approaches and 3D printing solutions.

Future works aim at integrating seamlessly the [E][D][S] iterations within a new multi-purpose open-source project integrating the safe-by-design approaches already developed by INRA (essentially 1D, multiple steps, multiple materials) with these new optimization strategies targeting environmental efficiency and fully 3D. The benefits are expected to be mutual and greater than the sum of the two parts. Mass transfer are integrated over the exact thickness profile of the bottle and surface areas are not anymore overestimated. Shelf-life can be estimated according to various criteria: weight loss, collapse, specific migration. In the case of PET bottles, the capacity to estimate the best ratio of virgin (high impact) and mechanically recycled (low impact) materials will be significant. In this particular case, current EU regulation requires a design and storage conditions, which would ensure a migration lower than 0.017 μg·kg^−1^ in the beverage for a residual post-consumer contamination of recycled PET conservatively set at 3 mg·kg^−1^ (Barthélémy et al., 2014). The chief difficulty at this stage is the absence of standardized templates and non-commercial licenses for food packaging design. Bringing the proper amount of plastics to specific food usages, supply chains, and consumer experiences could be accessible while minimizing health and environmental impacts.

## Author Contributions

YZ carried out the experiments and ran the simulations. YZ and OV wrote the computational codes and wrote the manuscript. BG reviewed and revised the manuscript. All authors designed the experiments, analyzed and discussed the results.

### Conflict of Interest Statement

YZ and BG were employed by the group Pernod-Ricard. The remaining author declares that the research was conducted in the absence of any commercial or financial relationships that could be construed as a potential conflict of interest.
